# Identifying the most important research, policy and practice questions for substance use, problematic alcohol use and behavioural addictions in autism (SABA-A): A priority setting partnership

**DOI:** 10.1016/j.comppsych.2023.152393

**Published:** 2023-05-15

**Authors:** Julia M.A. Sinclair, Betul Aslan, Roberta Agabio, Amith Anilkumar, Mark Brosnan, Ed Day, Nicki A. Dowling, Chelsey Flood, Jon E. Grant, Robyn Halliday, Björn Hofvander, Leesa Howes, Rachel Moseley, Bronwyn Myers, Vincent O'Connor, Gabriel Shaya, Shane Thomas, Janine Robinson, Samuel R. Chamberlain

**Affiliations:** 1 Faculty of Medicine, University of Southampton, UK; 2 Department of Biomedical Sciences, Section of Neuroscience and Clinical Pharmacology, University of Cagliari, Italy; 3 Neuroscience Institute, Section of Cagliari, National Research Council, Italy; 4 Centre for Applied Autism Research, University of Bath, UK; 5 Institute of Mental Health, University of Birmingham, UK; 6 School of Psychology, Deakin University, Geelong, Australia; 7 University of the West of England, UK; 8 Department of Psychiatry & Behavioral Neuroscience, University of Chicago, Pritzker School of Medicine, Chicago, IL, USA; 9 Expert by Experience, UK; 10 Department of Clinical Sciences, Faculty of Medicine, Lund University, Lund, Denmark; 11 Human Kind, UK; 12 Cognitive and Affective Neuroscience research group, Bournemouth University, UK; 13 Curtin enAble Institute, Faculty of Health sciences, Curtin University, Perth; 14 Alcohol, Tobacco and Other Drug Research Unit, South African Medical Research council; 15 Division of Addiction Psychiatry, Department of Psychiatry and Mental Health, University of Cape Town, Cape Town, South Africa; 16 School of Biological Sciences, University of Southampton, UK; 17 Turning Point, UK; 18 Federation University, Ballarat, VIC Australia; 19 Chitra Sethia Autism Centre, Cambridgeshire and Peterborough NHS Foundation Trust, Fulbourn, Cambridge, UK; 20 Southern Health NHS Foundation Trust, Southampton, UK

**Keywords:** Problematic alcohol use, substance use, gambling, autism, addiction

## Abstract

**Background:**

Autistic people are more likely to report problematic alcohol and other substance use when compared to the general population. Evidence suggests that up to one in three autistic adults may have an alcohol or other substance use disorder (AUD/SUD), although the evidence base for behavioural addictions is less clear. Autistic people may use substances or engage in potentially addictive behaviours as a means of coping with social anxiety, challenging life problems, or camouflaging in social contexts. Despite the prevalence and detrimental effects of AUD, SUD and behavioural addictions in community samples, literature focusing on the intersection between autism and these conditions is scarce, hindering health policy, research, and clinical practice.

**Methods:**

We aimed to identify the top 10 priorities to build the evidence for research, policy, and clinical practice at this intersection. A priority‐setting partnership was used to address this aim, comprising an international steering committee and stakeholders from various backgrounds, including people with declared lived experience of autism and/or addiction. First, an online survey was used to identify what people considered key questions about Substance use, alcohol use, or behavioural addictions in autistic people (SABA‐A). These initial questions were reviewed and amended by stakeholders, and then classified and refined to form the final list of top priorities via an online consensus process.

**Outcomes:**

The top ten priorities were identified: three research, three policy, and four practice questions. Future research suggestions are discussed.

## Introduction

There is not a universally accepted way to refer to autistic people, so in line with the general preference of many in the autistic community, ^1^ this project, in conjunction with stakeholders, agreed to use identity-first language. Autism spectrum conditions (hereafter autism) are a family of neurodevelopmental conditions affecting more than 1% of people worldwide ^2,3^ and are considered a major public health concern. ^4–6^ Compared to the general population, data indicate that autistic people are two times more likely to experience early mortality, and higher rates of co-occurring physical and mental health–related conditions. ^7–12^

Furthermore, autistic people are at increased risk of problematic alcohol, other substance use, and gambling-related harms (including gambling disorder) ^13–15^ particularly when there is co-occurring attention deficit hyperactivity disorder (ADHD) ^16,17^ or a family history of addictions. ^18^

In addition to alcohol and substance use disorders (AUD/SUD), gambling disorder is now the first ‘behavioural addiction’ to also be recognised in the Diagnostic and Statistical Manual Version 5 (DSM-5). This is in recognition that certain behaviours can also be reinforcing and lead to an addictive pattern of behaviours in some individuals. ^19^ Although gambling disorder is the only ‘formally recognised’ behavioural addiction in DSM-5, the International Classification of Diseases (ICD-11) does recognise other entities such as ‘gaming disorder’. ^20^ Therefore, the concept of behavioural addiction may extend beyond gambling into other types of repetitive activities that become habitual, hard to suppress, and result in functional impairment. ^21^

Single studies have shown higher baseline alcohol consumption is associated with higher levels of autistic traits in a treatment seeking population, ^22^ and elevated levels of autistic traits in a significant proportion of people seeking treatment for SUD. ^23^ Community samples also suggest an association between autistic traits with gambling, ^24^ and SUD. ^16^

However, there is also evidence to the contrary. For example, studies have shown that autism is not associated with poor or enhanced performance on the Iowa Gambling Task. ^25^ Additionally, the prevalences of SUDs (including tobacco smoking^26^) are significantly lower in autistic patients compared to non-autistic patients managed within inpatient psychiatric facilities, ^25,27,28^ as well as in an outpatient sample compared to controls. ^29^ Conflicting results are likely to be due to a number of reasons: the spectrum nature of autism; the spectrum of AUD, SUD and behavioural addictions; the cut-offs used in different studies; the lack of (and/or inconsistent use of) validated screening and diagnostic tools, and the use of these tools in co-occurring states. Therefore, although the most recent review in the area confirms the link between autism and SUD^30^ it remains unclear how these conditions interact and which autism-specific factors increase or decrease the risk of individuals developing AUD, SUD or behavioural addictions.

A recent large online survey comparing the self-reported substance use of autistic and non-autistic young people, found that autistic responders were less likely to use alcohol or substances, but when they did, were significantly more likely to be doing so as a coping strategy for a range of underlying behavioural and mental health difficulties. ^31^ The accompanying commentary noted with surprise the relative lack of research in this area. ^32^

Autistic adolescents and adults experience multiple barriers accessing primary healthcare services, ^33,34^ especially for those from minority backgrounds. ^35^ Similarly, access and uptake for addiction treatment is low across health settings, and globally the percentage of people with AUD who access treatment services is low. ^36^ Autistic individuals with addiction are thus likely to experience major barriers to accessing support and care, including perceived and enacted stigma. ^37,38^ It is therefore essential to address the mechanisms, prevalence, prognosis, treatment needs and successful interventions for the spectrum of SUD and behavioural addictions in autistic individuals to develop effective prevention strategies and to better support autistic people and their families to facilitate prevention, treatment and recovery.

Given the inconclusive research and lack of guidance for researchers, clinicians, and policymakers, we aimed to identify the key policy, research and clinical practice questions that should be asked about Substance use, Alcohol and Behavioural Addictions in Autism (hereafter referred to as ‘SABA-A’) using a consensus exercise. We also sought to rank these priorities to guide future policymakers, researchers, and practitioners on the most urgent issues on which to work.

## Methods

Priority Setting Partnerships (PSPs) are widely used in healthcare policy settings to identify uncertainties in various health‐related subjects, and to reach a consensus on the most important policy, research, and clinical practice priorities (e.g. preterm birth, ^39^ dementia, ^40^ and alcohol-related liver disease^41^). The five-stage process defined by the James Lind Alliance (JLA) guideline^42^ was modified to facilitate the engagement of a wider range of stakeholders, to identify policy (including health) and practice priorities in addition to clinical research priorities and to focus at the intersection of autism with substance use and behavioural addictions rather than autism more specifically. ^43^ All abbreviations are defined in the supplementary material (Appendix 1).

### Stage 1: Establishing the PSP

#### Stakeholders

The PSP consisted of establishing an international steering committee (N=10) and inviting stakeholders to be involved in the project and participate in the consensus exercise. The project was advertised widely, including through the project website^44^ and social media, ^45^ and relevant information was hosted by the Society for the Study of Addiction (SSA). The SSA website has over 150,000 page views per year from over 60,000 website users. It is accessed by people in over 150 countries, although it is predominantly accessed by people in the UK, US, Australia and Canada. Stakeholders in all jurisdictions were encouraged to participate in the project, and further disseminate the request for involvement. Potential stakeholders were defined as ‘any person who has an interest in autism and/or addictions and wishes to be part of the SABA-A project’. Stakeholders were not required to give any demographic information, but in the subset who participated in the online survey (N=78), 31% (n=22) identified their primary interest as being an autistic person with lived experience of addictions (including family members), 14% (n=10) as healthcare professionals (including autistic individuals) working in the field of autism or addiction, 19% (n=14) as public organisation representatives, 26% (n=19), as researchers with and interest in the area of autism or addiction and 10% (n=7), as other people who are interested in the intersection between those conditions, but who did not self‐identify in any of the above categories. When people identified with more than one category, they were included in all relevant categories.

#### Steering committee

The project steering committee constituted an international group of 10 experts, from the UK, USA, Portugal, and Italy. The group was structured to include people with lived experience (of autism and/or addiction[s]), autism experts, and addiction researchers. The steering committee met four times over the 18‐month project and its purpose was to broaden the reach of stakeholder engagement, give feedback on the initial and ongoing scoping reviews, and be part of the consensus process. The group determined the scope of the project (the intersection between substance use, alcohol use and behavioural addictions in autistic people). After an initial scope of the literature Foetal Alcohol Spectrum Disorder (FASD) was also included. The challenge around terminology was a key task for the steering group. ‘Autism’, ‘alcohol consumption’, ‘substance use’ and ‘gambling’ are all spectrum concepts from the normative to highly disabling, and potential behavioural addictions are similarly broad. Terminology has changed and been redefined over time, and terms are used variably and interchangeably in the literature. As there is no universally accepted way to describe autism, the steering group agreed on identity‐first language, and the use of terms ‘problematic alcohol use’, ‘substance use’ and ‘gambling’ as the motif for these potentially addictive conditions. It was also recognised that there was a diversity of opinion around what might constitute acceptable, culturally sensitive and non-stigmatising language given the diversity of the stakeholder group that was part of the process, and this was kept under review over the course of the project.

### Stage 2: Gathering the scientific information: Literature reviews

Three literature reviews were undertaken by the project team to review the evidence base on problematic alcohol use and autism; substance use and autism; and behavioural addictions and autism. These reviews aimed to scope the evidence base at the intersection between autism and addictions, as part of identifying where the gaps in the literature might be. After key search terms were identified from previous literature and reviewed by the steering committee, a database search was conducted on PUBMED under these three domains. A list of the search terms and reviewed papers are in the supplementary material (Appendix 2). Detailed results from the literature reviews are outside the scope of the current manuscript and will be reported separately. ^46^ However, they highlighted the lack of studies focusing on addictions in autism, and that the available studies, showed mixed results and were rarely replicated.

Results were mixed due to the differences in the autism and addiction definitions, sample characteristics, screening/inclusion methods and measurements used in identified studies. A summary of the findings was discussed by the steering group and it was concluded that there was no robust evidence on any of the areas under consideration that would exclude any area from further consideration.

### Stage 3: Identifying questions (online survey)

As part of a PSP, stakeholders are surveyed with the request to submit any questions that they think should be a priority for future research. This is a key part of stakeholder engagement, rather than research and so an ethical opinion was not required. The survey was launched online in October 2021 – and remained open for 13 weeks using the software SurveyMonkey (full details are provided in supplementary material Appendix 3). Participants were encouraged to take the survey and submit any potential research questions they had about the intersection between AUD, SUD, or behavioural addictions, and autism based on the James Lind Template.

Following the online survey, and initial analysis of responses, additional workshops were held to identify any additional questions/areas not covered in the initial survey responses, including around specific marginalised groups who may not have engaged with the survey (e.g., minority ethnic groups, homeless populations, gender and sexual identity etc.). These additional online workshops also facilitated the modification and clarification of questions, and the language used to express them. All the raw data are provided in supplementary material Appendix 4).

### Stage 4: Refining and classifying the questions about evidence uncertainties

**Figure 1:**
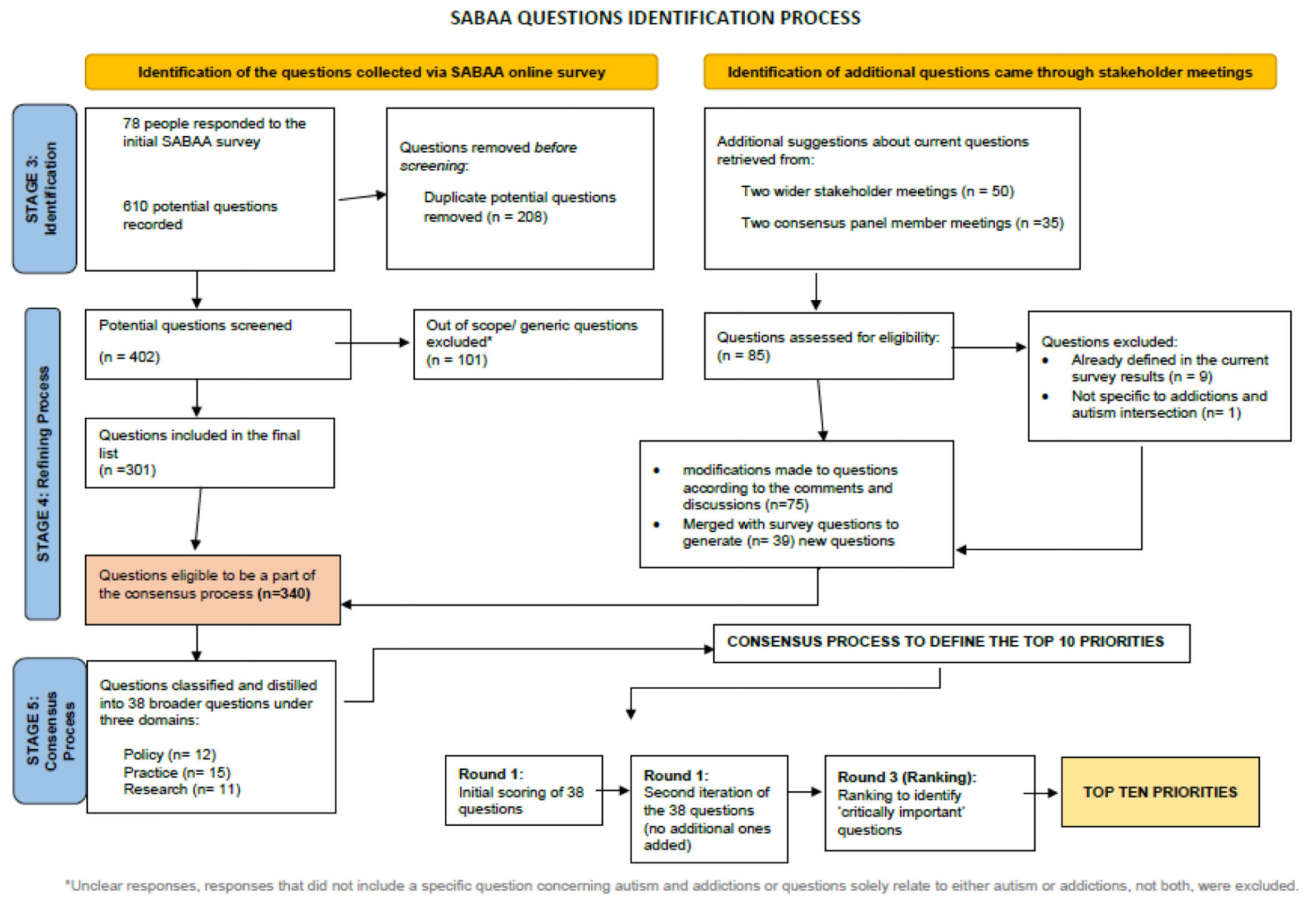
Process for identification of consensus questions

**Figure 2:**
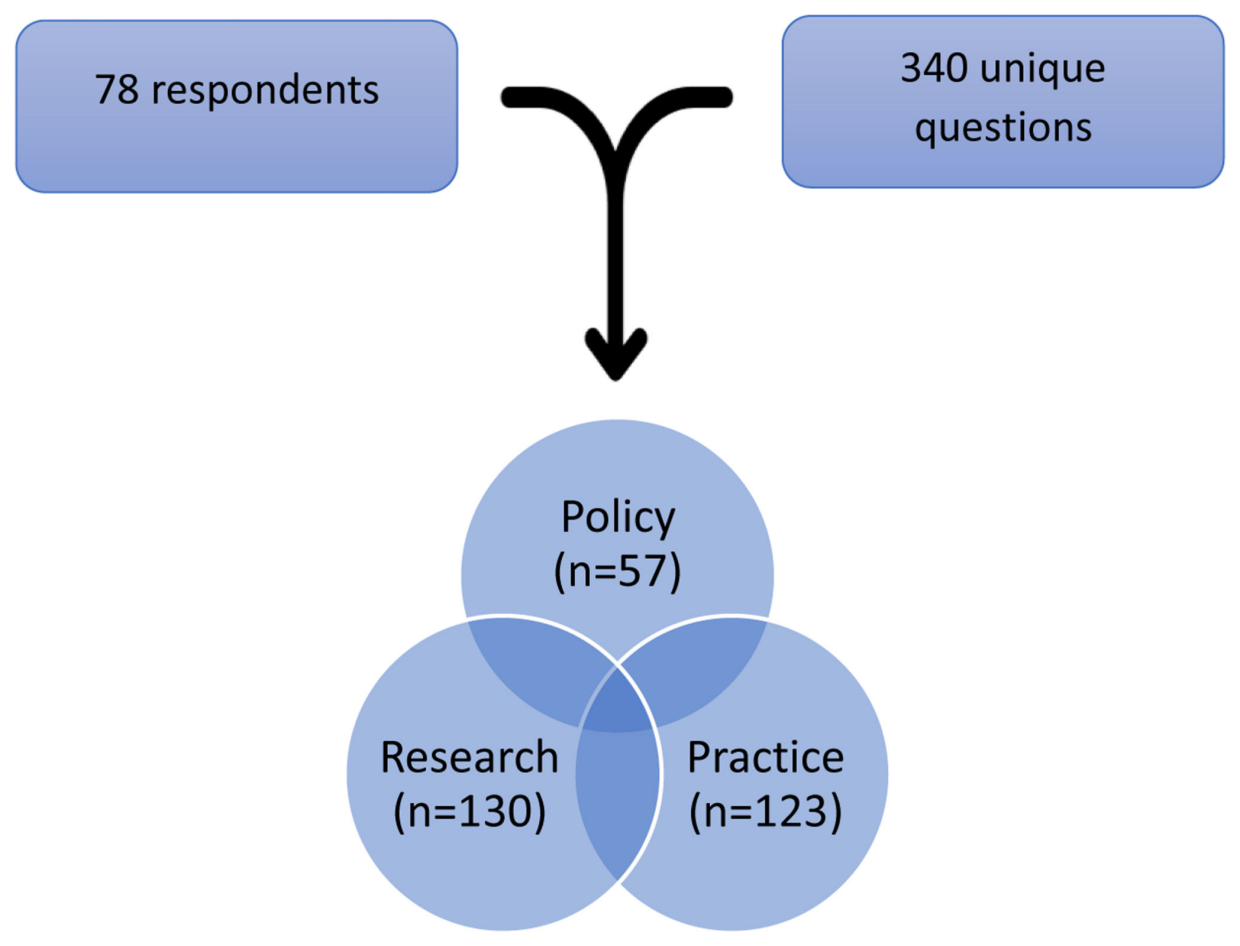
Final distribution of questions by category

All questions (responses) submitted by the stakeholders via the survey or during stakeholder consultations were included in the first stage of analysis. Duplicate and out of scope questions (i.e. those that did not pertain specifically to the intersection of AUD, SUD or behavioural addictions in autism) were then excluded, and remaining questions were defined as being eligible for the consensus process (Figure 1). Categorisation of the raw data went through several iterations. NVivo software was used for thematic analysis and three overarching categories of questions were identified (Figure 2).

### Stage 5: Consensus process: Reducing the questions and identifying the Top‐10 priorities

After necessary ethical approvals were obtained (ERGO 67084.A1.R), potential panel members for the consensus process, drawn from the wider stakeholder group, were invited to participate in an online consensus process.

The panel consisted of 22 researchers, clinicians, and people with diverse lived experience (including autism and addictions) (these were not mutually exclusive categories). Contributors came from the UK (n= 14), Australia (n=3), Europe (n=2), and the USA (n=2). Online consensus processes facilitate international engagement, and have been found to be effective for synthesising the views of autistic individuals with researchers and practitioners. ^47^

The raw data (see Figure 1) were further analysed and classified into question themes. Similar questions were brought together to form comprehensive ‘topic questions’. This classification and re-creation process was discussed during stakeholder meetings and with the consensus panel group to refine the principles and scope and language used.

The final list of ‘Topic Questions’ went forward into the consensus process. An online platform (DelphiManager) was used to host the questions for the virtual iterations of the consensus process.

The rating process consisted of two iterative rounds and a subsequent ranking round. Each round was open for a two-week window and respondents were sent reminders to reply. In the first-round participants were provided with the final 38 ‘topic questions’. They were asked to rate each question on a 9-point scale from 1 to 9, with 1-3 indicating ‘not important’; 4-6 indicating ‘important but not critical’ and 7-9 indicating ‘critical’. Panel members were also given the option to choose ‘unable to rate’ if they believed a particular question was not suitable to rate for any reason. They were also given the chance to suggest additional questions, provide feedback on any of the questions, or explain their reason for giving a particular rating.

During the second round, panel members were provided with the information of their previous ratings, as well as other members’ ratings (as percentages). They were asked to indicate their final ratings for each question on the same scoring from 1-9. They were further given the option to provide explanation/feedback on any significant changes to their ratings.

Questions with the highest consensual rating (e.g., rated critical (rated 7,8 or 9) by 70% or more of panel members) were included in the final (ranking) round. During this round panel members decided on the final list of priorities by ranking the importance of questions in order. The total ranking score was taken as an indicator of priority. Top-10 priorities were defined at the end of the process.

### Role of the funding source

This Priority Setting Partnership was funded by a grant from the Society for the Study of Addiction (SSA). The SSA did not have any involvement in the study design, collection, analysis and interpretation of data, in the writing of the final report, or in the decision to submit the paper for publication.

## Results

### Stage 3: Identifying questions (online survey)

Overall, 78 respondents participated in the on-line survey.

Composition of the stakeholders by groups showed that 28% of the participants identified themselves as a ‘researcher, healthcare professional, professional or volunteer who works with people with addictions’; 24% as a ‘researcher, healthcare professional, professional or volunteer who works with autistic individuals’; 33% as ‘autistic’; 22% as ‘a person who has personal experience of substance, alcohol, or behavioural addiction (now or previously)’ and 19% as ‘a family member/carer/friend for an autistic individual with substance, alcohol, or behavioural addiction (now or previously)’, and 14% as other. Responses were not mutually exclusive.

A total of 610 responses (potential questions to prioritise) were submitted. After screening, 309 questions were excluded due to being out of scope of the PSP, being too vague, or constituting duplication of other questions. This resulted in 301 unique questions.

Additional stakeholder workshops in which the survey results were discussed generated 85 further potential questions. A significant number of these were not covered in the initial online survey questions, hence they were included as new additional questions. At the end of this process, a total of 340 questions were identified (see Figure 1 for a detailed explanation of the process, and Appendix 3; supplementary material for the list of all 340 questions).

### Stage 4: Refining and classifying the questions about evidence uncertainties

Categorisation of the raw data over several iterations resulted in three broad types of questions: policy, research, and practice. These categories and example questions were discussed during the stakeholder and consensus panel meetings (see Figure 2).

### Stage 5: Consensus process: Reducing the questions and identifying the Top–10 priorities

A final over inclusive list of 38 ‘topic questions, was submitted to the consensus process. (See Figure 2).

Participants rated each priority from one to nine in the first two rounds and then ranked the top 14 priorities in the last ranking round. Sum scores were taken as the indicator of consensus in the final round. The top-10 priorities concerning policy, practice and research are given in Table 1.

## Discussion

The nature and aetiology of addictions in autistic people, including the contribution and importance of co-occurring physical and mental health conditions, has been little studied in the literature and the results are mixed. ^12,48,49^ We conducted a PSP to identify the top10 priorities for health policy, research, and clinical practice. This represents a vital first step in building a more systematic evidence base, which will help to advance the field more effectively. Our PSP had extensive contributions from international stakeholders with diverse backgrounds, including people with lived experience of being autistic and/or living with addictions. A consensus process was conducted to agree the most important priorities. We identified top priorities in three different domains of policy (n=3), practice (n=4) and research (n=3).

In terms of the research domain, a lack of knowledge around the specific triggers, risk factors or facilitators for autistic people to develop substance, alcohol and/or behavioural addictions (SABA) was highlighted as a key area of uncertainty and was ranked as the highest priority by this PSP. To develop effective prevention strategies, more needs to be understood about the mechanisms that impact on developing alcohol, substance or gambling problems in autistic individuals as well as whether any autism-related features may be protective. The other research domains highlighted as most important included investigation of the impact that other conditions in autistic people (e.g. ADHD, OCD) may have on the development and maintenance of addictive patterns of behaviour, and an understanding of how neural pathways and executive functioning might differ in autistic people with SABA compared with other groups.

In terms of health policy domains, stigma was considered a key topic. In addition to the responses submitted as part of the initial online survey, stigma, and ableism, as well as their effects on autistic people in relation to potential addictive behaviour were discussed in detail during the stakeholder workshop. Given that autistic people and those with addictions are frequently marginalised, steps to reduce ableism and stigma by enhancing awareness and acceptance were ranked as a key policy priority. The other two policy domains listed in the top-10 list of priorities comprise the training of professionals to enable them to better support autistic people with SABA, ^50^ and adaptations of screening/diagnostic tools to enhance their utility for the diagnosis of SABA in autistic people, as per parallel efforts to support better detection of anxiety^51^, depression^52^ and suicidal ideation^53^ in autistic people. These priorities may substantially contribute to improving diagnosis and treatment of autistic people with SABA.

Under the clinical practice domain, four priorities were ranked in the top-10. Given the nature of autistic anxiety and difficulties around communicating with non-autistic others, ^54^ individual therapies seem to work better for autistic individuals than group-based approaches. ^55^ To achieve effective results, SUD treatment providers may benefit from further training in how to work effectively with autistic individuals with SABA. ^56,57^ It is suggested that adjustments tailored to autistic needs should be considered to reduce risk of attrition, enhance engagement in treatment and improve treatment outcomes. ^57,58^

An important limitation of the existing evidence base is that although there is some evidence about physical and mental health-related co-occurring conditions in autistic people with SABA, it is not clear which other conditions might contribute to the development or maintenance of addictions in autistic people. Given high rates of ADHD, anxiety, depression, sleep disorders and intellectual disability in this population, ^48,59^ research into underlying causal pathways for the development of SABA in autistic people is essential.

The strength of this PSP lies in its involvement of a diverse range of contributors, bringing a wealth of perspectives. Significant efforts were made to adapt the PSP tools used by the James Lind Alliance to make them accessible to a diverse stakeholder group and widen the remit to include policy and practice questions. As with all such processes, it is limited to the input of those people who chose to engage with the process. However, the final contributors do represent an international group with a wide range of experience between them. A limitation of the current approach is that the manualised nature of question selection and topic distillation could have led to bias. Future work could recruit larger panels of experts to collect data and distil topics using other methodological approaches – such as factor analysis, or Latent Dirichlet Allocation (LDA). These methods were not considered appropriate here due to the sample size.

In conclusion, substance, alcohol and behavioural addictions in autistic people is an area that has had limited consideration in the research, policy and practice arenas. This PSP was challenged to limit the number of areas to those that were considered ‘critical’ given the large number of unanswered questions that remain and the impact this has on the lives of individuals affected and their circles of concern. This PSP has identified key priorities for research, policy, and practice, to facilitate the much needed evidence base in this area.

## Supplementary Material

Supplementary material

## Figures and Tables

**Figure 3:**
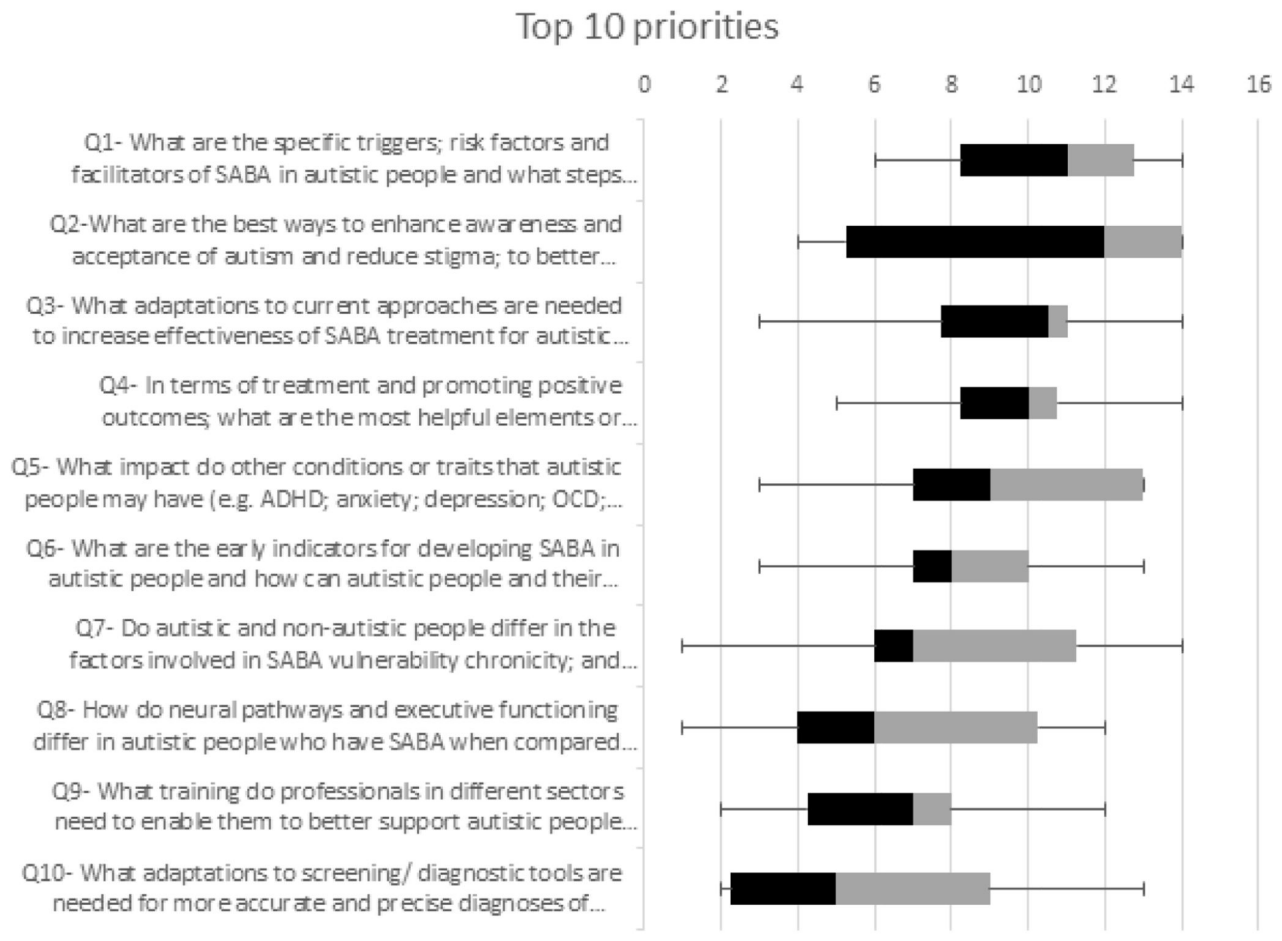
Box and whisker plots of the top 10 priorities, comparison of the distribution of the ratings, the central boxes represent 25 to 75 percentiles.

**Table 1:** Top-10 priorities

Order	Top 10 priorities	Domain	TRS	Mean Score	Median Score	Score Range	SD
1	What are the specific triggers; risk factors and facilitators of SABA in autistic people and what steps could be taken to prevent them?	Research	191	10·61	11	6-14	2·64
2	What are the best ways to enhance awareness and acceptance of autism and reduce stigma; to better support autistic people to promote positive outcomes or prevent SABA?	Policy	178	9·89	12	4-14	4·42
3	What adaptations to current approaches are needed to increase effectiveness of SABA treatment for autistic people?	Practice	171	9·50	10.5	3-14	3·03
4	In terms of treatment and promoting positive outcomes; what are the most helpful elements or approaches in the management of SABA in autistic people?	Practice	170	9·44	10	5-14	2·68
5	What impact do other conditions or traits that autistic people may have (e.g., ADHD; anxiety; depression; OCD; impulse control etc.) on the development and maintenance of SABA?	Research	169	9·39	9	3-13	3·48
6	What are the early indicators for developing SABA in autistic people and how can autistic people and their families identify these indicators?	Practice	151	8·39	8	3.13	2·85
7	Do autistic and non-autistic people differ in the factors involved in SABA vulnerability chronicity; and response to treatment?	Practice	146	8·11	7	1-14	3·98
8	How do neural pathways and executive functioning differ in autistic people who have SABA when compared to autistic people who do not have SABA and non-autistic people?	Research	119	6·61	6	1-12	3·81
9	What training do professionals in different sectors need to enable them to better support autistic people who have SABA?	Policy	118	6·56	7	2-12	3·01
10	What adaptations to screening/diagnostic tools are needed for more accurate and precise diagnoses of SABA in autistic people?	Policy	114	6·33	5	2-13	4·33

SABA, Substance alcohol and/or behavioural addictions; ADHD, Attention Deficit Hyperactivity Disorder; OCD, Obsessive Compulsive Disorder; TRS: Total ranking score; SD, Standard Deviation *Priorities are ranked according to their total rating scores in the last ranking round, 1^st^ priority represents the most important/critical question and the degree decreases downwards.

## Data Availability

All raw data from the online survey is presented in the supplementary materials.

## Highlights

- Little is known about the overlap between autism and addiction, yet both are common
- This priority setting partnership (PSP) and consensus study identified the top research, policy, and clinical practice questions regarding this overlap
- Identification of these priorities will assist researchers and other experts, funders, and policy makers to address the most relevant knowledge gaps.

## References

[1] Kenny L, Hattersley C, Molins B, Buckley C, Povey C, Pellicano E (2016). Which terms should be used to describe autism? Perspectives from the UK autism community. Autism.

[2] Zeidan J, Fombonne E, Scorah J (2022). Global prevalence of autism: A systematic review update. Autism Res.

[3] Maenner MJ, Shaw KA, Bakian AV (2021). Prevalence and Characteristics of Autism Spectrum Disorder Among Children Aged 8 Years - Autism and Developmental Disabilities Monitoring Network, 11 Sites, United States, 2018. Mmwr SurveillSumm.

[4] Leigh JP, Du J (2015). Brief Report: Forecasting the Economic Burden of Autism in 2015 and 2025 in the United States. Journal of Autism and Developmental Disorders.

[5] Newschaffer CJ, Curran LK (2003). Autism: An emerging public health problem. Public Health Rep.

[6] McCall B (2017). UK failing to meet the needs of people with autism. Lancet.

[7] Lai MC, Kassee C, Besney R (2019). Prevalence of co-occurring mental health diagnoses in the autism population: a systematic review and meta-analysis. Lancet Psychiatry.

[8] Roy M, Prox-Vagedes V, Ohlmeier DM, Dillo W (2015). Beyond childhood: psychiatric comorbidities and social background of adults with Asperger syndrome. Psychiatria Danubina.

[9] Schendel DE, Overgaard M, Christensen J (2016). Association of Psychiatric and Neurologic Comorbidity With Mortality Among Persons With Autism Spectrum Disorder in a Danish Population. JAMA Pediatr.

[10] Bilder D, Botts EL, Smith KR (2013). Excess mortality and causes of death in autism spectrum disorders: a follow up of the 1980sUtah/UCLA autism epidemiologic study. J Autism Dev Disord.

[11] Chien YL, Wu CS, Tsai HJ (2021). The Comorbidity of Schizophrenia Spectrum and Mood Disorders in Autism Spectrum Disorder. Autism Res.

[12] Simonoff E, Pickles A, Charman T, Chandler S, Loucas T, Baird G (2008). Psychiatric disorders in children with autism spectrum disorders: prevalence, comorbidity, and associated factors in a population-derived sample. J Am Acad Child Adolesc Psychiatry.

[13] Umeda M, Shimoda H, Miyamoto K (2019). Comorbidity and sociodemographic characteristics of adult autism spectrum disorder and attention deficit hyperactivity disorder: epidemiological investigation in the World Mental Health Japan 2nd Survey. Int J Dev Disabil.

[14] Roy M, Prox-Vagedes V, Ohlmeier MD, Dillo W (2015). Beyond childhood: psychiatric comorbidities and social background of adults with Asperger syndrome. Psychiatr Danub.

[15] Kaltenegger HC, Doering S, Gillberg C, Wennberg P, Lundstrom S (2021). Low prevalence of risk drinking in adolescents and young adults with autism spectrum problems. Addict Behav.

[16] De Alwis D, Agrawal A, Reiersen AM (2014). ADHD symptoms, autistic traits, and substance use and misuse in adult Australian twins. J Stud Alcohol Drugs.

[17] Solberg BS, Zayats T, Posserud MB (2019). Patterns of Psychiatric Comorbidity and Genetic Correlations Provide New Insights Into Differences Between Attention-Deficit/Hyperactivity Disorder and Autism Spectrum Disorder. Biol Psychiatry.

[18] Sundquist J, Sundquist K, Ji J (2014). Autism and attention-deficit/hyperactivity disorder among individuals with a family historyof alcohol use disorders. Elife.

[19] American Psychiatric Association (2013). Diagnostic and statistical manual of mental disorders.

[20] World Health Organization (2019). ICD-11: International classification of diseases (11th revision).

[21] Grant JE, Chamberlain SR (2016). Expanding the definition of addiction: DSM-5 vs. ICD-11. CNS Spectrums.

[22] Hildebrand Karlen M, Stalheim J, Berglund K, Wennberg P (2021). Autistic Personality Traits and Treatment Outcome for Alcohol Use Disorders. J Nerv Ment Dis.

[23] McKowen J, Woodward D, Yule AM (2022). Characterizing autistic traits in treatment-seeking young adults with substance use disorders. Am J Addict.

[24] Grant JE, Chamberlain SR (2020). Autistic traits in young adults who gamble. CNS Spectr.

[25] Zeif D, Yechiam E (2020). Autism is not associated with poor or enhanced performance on the Iowa Gambling Task: A Meta-Analysis. Neurosci Biobehav Rev.

[26] Bejerot S, Nylander L (2003). Low prevalence of smoking in patients with autism spectrum disorders. Psychiatry Res.

[27] Esan F, Chester V, Gunaratna IJ, Hoare S, Alexander RT (2015). The clinical, forensic and treatment outcome factors of patients with autism spectrum disorder treated in a forensic intellectual disability service. J Appl Res Intellect Disabil.

[28] Mandell DS, Lawer LJ, Branch K (2012). Prevalence and correlates of autism in a state psychiatric hospital. Autism.

[29] Yule AM, DiSalvo M, Biederman J (2021). Decreased risk for substance use disorders in individuals with high-functioning autism spectrum disorder. Eur Child Adolesc Psychiatry.

[30] Ressel M, Thompson B, Poulin M-H (2020). Systematic review of risk and protective factors associated with substance use and abuse inindividuals with autism spectrum disorders. Autism.

[31] Weir E, Allison C, Baron-Cohen S (2021). Understanding the substance use of autistic adolescents and adults: a mixed-methods approach. The Lancet Psychiatry.

[32] Livingston LA (2021). Substance use, coping, and compensation in autism. The Lancet Psychiatry.

[33] Doherty AJ, Atherton H, Boland P (2020). Barriers and facilitators to primary health care for people with intellectual disabilities and/or autism: an integrative review. BJGP Open.

[34] Ghanouni P, Hood G, Weisbrot A, McNeil K (2021). Utilization of health services among adults with autism spectrum disorders: Stakeholders' experiences. Res Dev Disabil.

[35] Slade G (2014). Diverse perspectives: The challenges for families affected by autism from black, Asian and Minority Ethnic communities.

[36] Gilchrist G, Moskalewicz J, Nutt R (2014). Understanding access to drug and alcohol treatment services in Europe: A multi-country service users’ perspective. Drugs: Education, Prevention and Policy.

[37] Kilian C, Manthey J, Carr S (2021). Stigmatization of people with alcohol use disorders: An updated systematic review of population studies. Alcohol Clin Exp Res.

[38] Link BG, Phelan JC (2006). Stigma and its public health implications. The Lancet.

[39] Duley L, Uhm S, Oliver S (2014). Top 15 UK research priorities for preterm birth. Lancet.

[40] Kelly S, Lafortune L, Hart N (2015). Dementia priority setting partnership with the James Lind Alliance: using patient and public involvement and the evidence base to inform the research agenda. Age Ageing.

[41] Alliance James Lind (2016). Alcohol-related Liver Disease.

[42] Alliance James Lind (2021). The James Lind Alliance Guidebook James Lind Alliance.

[43] Alliance James Lind (2016). Autism Top 10.

[44] Society for the Study of Addiction (2022). SABAA: Substance use, Alcohol and Behavioural Addictions in Autism.

[45] @PspSabaa (2021). Understanding the links between autism, addictive behaviour and substance use disorders-APriority Setting Partnership sabaa_psp.

[46] Chamberlain SR, Aslan B, Quinn A (2023). Autism and gambling: A systematic review, focusing on neurocognition. Neuroscience & Biobehavioral Reviews.

[47] Zervogianni V, Fletcher-Watson S, Herrera G (2020). A framework of evidence-based practice for digital support, co-developed with and for the autism community. Autism.

[48] Leader G, Moore R, Chen JL (2021). Attention deficit hyperactivity disorder (ADHD) symptoms, comorbid psychopathology, behaviour problems and gastrointestinal symptoms in children and adolescents with autism spectrum disorder. Ir J Psychol Med.

[49] Rydzewska E, Dunn K, Cooper SA (2021). Umbrella systematic review of systematic reviews and meta-analyses on comorbid physical conditions in people with autism spectrum disorder. Br J Psychiatry.

[50] Doherty M, Neilson S, O'Sullivan J (2022). Barriers to healthcare and self-reported adverse outcomes for autistic adults: a cross-sectional study. BMJ Open.

[51] Rodgers J, Farquhar K, Mason D (2020). Development and Initial Evaluation of the Anxiety Scale for Autism-Adults.

[52] Cassidy SA, Bradley L, Cogger-Ward H, Rodgers J (2021). Development and validation of the suicidal behaviours questionnaire-autism spectrum conditions in a community sample of autistic, possibly autistic and non-autistic adults. Mol Autism.

[53] Cassidy S, Bradley L, Cogger-Ward H, Graham J, Rodgers J (2021). Development and Validation of the Autistic Depression Assessment Tool-Adult (ADAT-A) in Autistic Adults.

[54] Davis R, Crompton CJ (2021). What Do New Findings About Social Interaction in Autistic Adults Mean for Neurodevelopmental Research?. Perspect Psychol Sci.

[55] Cooper K, Loades ME, Russell AJ (2018). Adapting Psychological Therapies for Autism - Therapist Experience, Skills and Confidence. Res Autism Spectr Disord.

[56] Helverschou SB, Brunvold AR, Arnevik EA (2019). Treating Patients With Co-occurring Autism Spectrum Disorder and Substance Use Disorder: A Clinical Explorative Study. Subst Abuse.

[57] Lalanne L, Weiner L, Trojak B, Berna F, Bertschy G (2015). Substance-use disorder in high-functioning autism: clinical and neurocognitive insights from two case reports. BMC Psychiatry.

[58] Brosnan M, Adams S (2022). Adapting Drug and Alcohol Therapies for Autistic Adults. Autism in Adulthood.

[59] Mutluer T, Aslan Genc H, Ozcan Morey A (2022). Population-Based Psychiatric Comorbidity in Children and Adolescents With Autism Spectrum Disorder: A Meta-Analysis. Front Psychiatry.

