# Supplementary material for: Identifying the most important research, policy and practice questions for substance use, problematic alcohol use and behavioural addictions in autism (SABA-A): A priority setting partnership
Source: Compr Psychiatry. Author manuscript; Available in PMC 2023 May 31. (PMC7614597; doi:10.1016/j.comppsych.2023.152393)
Supplement: Supplementary material [file EMS175735-supplement-Supplementary_material.docx]

**Identifying the most important research, policy and practice questions for substance use, problematic alcohol use and behavioural addictions in autism (SABA-A):**

**A priority setting partnership**

**Supplementary Material**

**Appendix 1: Abbreviations** **p2**

**Appendix 2: Literature Review search terms**  **p 3 - 5**

**Appendix 3: Online Survey** **p 6 – 10**

**Appendix 4: Raw data** **p 11- 30**

**Appendix 1: ABBREVIATIONS**

*AA: Alcoholics Anonymous*

*ACT: Acceptance and Commitment Therapy*

ADD: Attention Deficit Disorder

ADHD: Attention Deficit Hyperactivity Disorder

ASD: *Autism Spectrum Disorder*

ASSIST: Alcohol, Smoking and Substance Involvement Screening Test

AUD: Alcohol Use Disorder

BAME: Black, Asian, and Minority Ethnic

CAMHs: Child and Adolescent Mental Health Services

CBT: Cognitive Behavioural Therapy

DSM-5: Diagnostic and Statistical Manual Version 5

FASD: Foetal Alcohol Spectrum Disorder

GP: *General Practitioner*

ICD-11: International Classification of Diseases

*ID: Identify*

ITEP: International Treatment Effectiveness Project

JLA: James Lind Alliance

MDMA: 3,4-methylenedioxy-methamphetamine

NHS: the National Health Service

OCD: Obsessive Compulsive Disorder

PSP: Priority Setting Partnerships

## PSI: Peer Pressure Influence

PTSD: *Post-traumatic stress disorder*

SABA-A: Substance ,Alcohol, and Behavioural Addictions in Autism

SBIRT: *Screening, Brief Intervention and Referral to Treatment*

SD: Standard Deviation

SSA: Society for the Study of Addiction

SUD: Substance Use Disorder

TRS: Total ranking score

**Appendix 2: Literature Review search terms**

**Autistic spectrum and gambling search string:**

**Template for search strategy:**

Autism terms based on:

1. *Mobile device applications and treatment of autism spectrum disorder: a systematic review and meta-analysis of effectiveness*

Gambling terms based on :

1. *The use of self-management strategies for problem gambling: a scoping review*

Autism (OR):

1. Autis*
2. Asperger*
3. child development* disorder*
4. pervasive development*
5. infantile-autis*
6. Kanner-syndrom*
7. **Child Development Disorders, Pervasive (MesH)**


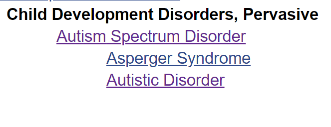


*(Included subcategories shown)*

1. neurodevelopment*

**AND**

Gambling (OR):

1. Gambl*
2. Bet*
3. casino*.tw,kf.
4. slot machine*.tw,kf.
5. lotter*
6. bingo
7. poker
8. scratch ticket*
9. scratch card*
10. electronic gaming machine*
11. craps
12. roulette
13. blackjack
14. **Gambling (MesH**

**Search on pubmed:**

((Autis*) OR (Asperger*) OR (child development* disorder*) OR (pervasive development*) OR (infantile-autis*) OR (Kanner-syndrom*) OR (Child Development Disorders, Pervasive[MeSH Major Topic])) AND ((Gambl*) OR (Bet*) OR (casino*) OR (slot machine*) OR (lotter*) OR (bingo) OR (poker) OR (scratch ticket*) OR (scratch card*) OR (electronic gaming machine*) OR (craps) OR (roulette) OR (blackjack) OR (Gambling[MeSH Major Topic]))

***Update (3.6.21) , adding (neurodevelopment*):***

((Autis*) OR (Asperger*) OR (child development* disorder*) OR (pervasive development*) OR (infantile-autis*) OR (Kanner-syndrom*) OR (neurodevelopment*) OR (Child Development Disorders, Pervasive[MeSH Major Topic])) AND ((Gambl*) OR (Bet*) OR (casino*) OR (slot machine*) OR (lotter*) OR (bingo) OR (poker) OR (scratch ticket*) OR (scratch card*) OR (electronic gaming machine*) OR (craps) OR (roulette) OR (blackjack) OR (Gambling[MeSH Major Topic]))

**RESULTS:**

Yield 343 results on pubmed, of which 9 were eligible and included (after double screening)

Submitted for publication

**Autistic spectrum and substance use search string:**

**Template for search strategy:**

Terms based on:

- Lewer, D., Freer, J., King, E., Larney, S., Degenhardt, L., Tweed, E. J., ... & Morley, K. I. (2020). Frequency of health‐care utilization by adults who use illicit drugs: a systematic review and meta‐analysis. *Addiction*, *115*(6), 1011-1023.
- Jones, A., Remmerswaal, D., Verveer, I., Robinson, E., Franken, I. H., Wen, C. K. F., & Field, M. (2019). Compliance with ecological momentary assessment protocols in substance users: a meta‐analysis. *Addiction*, *114*(4), 609-619.
- Magill, M., Ray, L., Kiluk, B., Hoadley, A., Bernstein, M., Tonigan, J. S., & Carroll, K. (2019). A meta-analysis of cognitive-behavioral therapy for alcohol or other drug use disorders: Treatment efficacy by contrast condition. *Journal of consulting and clinical psychology*, *87*(12), 1093.

| **Substance misuse OR**  chemical dependenc*  substance misuse*  substance abus*  substance use  drug user*  drug abus*  drug dependen*  inject* drug*  heroin  opiate*  cocaine  crack  amphetamine*  methamphetamine  benzodiazepine  mdma  ecstasy  cannabis  Substance-Related Disorders  Amphetamine-Related Disorders  Cocaine-Related Disorders  Heroin Dependence  Substance Abuse  Marijuana abuse  Smoking  Cigarette  Tobacco  Stimulant  Illicit drug  Nicotine  Caffeine | **AND** | **Autism (OR):**     - Autis* - Asperger* - Pervasive developmental disorder - ASD - PDD - Kanner - Neurodevelopmental - Child development disorders, pervasive (MesH) |
| --- | --- | --- |

**Search string:**

“Substance misuse”[Title/Abstract] OR “chemical dependenc*”[Title/Abstract] OR “substance misuse*”[Title/Abstract] OR “substance abus*”[Title/Abstract] OR “substance use”[Title/Abstract] OR “drug user*”[Title/Abstract] OR “drug abus*”[Title/Abstract] OR “drug  dependen*”[Title/Abstract] OR “inject* drug*”[Title/Abstract] OR heroin[Title/Abstract] OR opiate*[Title/Abstract] OR cocaine[Title/Abstract] OR crack[Title/Abstract] OR amphetamine*[Title/Abstract] OR methamphetamine[Title/Abstract] OR benzodiazepine[Title/Abstract] OR mdma[Title/Abstract] OR ecstasy[Title/Abstract] OR cannabis[Title/Abstract] OR “Substance-Related Disorders"[Mesh] OR “Amphetamine-Related Disorders"[Mesh] OR “Cocaine-Related Disorders"[Mesh] OR “Heroin Dependence"[Mesh] OR “Marijuana abuse"[Mesh] OR Smoking[Title/Abstract] OR Cigarette[Title/Abstract] OR Tobacco[Title/Abstract] OR Stimulant[Title/Abstract] OR “Illicit drug"[Title/Abstract] OR “Nicotine”[Title/Abstract] OR “Caffeine”[Title/Abstract]

**AND**

"Child Development Disorders, Pervasive"[Mesh] OR Autis*[Title/Abstract] OR Asperger*[Title/Abstract] OR “Pervasive developmental disorder” [Title/Abstract] OR ASD[Title/Abstract] OR PDD[Title/Abstract] OR Kanner[Title/Abstract] OR “Neurodevelopmental Disorders”[Title/Abstract]

**RESULTS:**

771 339 hits for **substance use terms**

73 491 hits for **autism terms**

1359 hits for **‘autism’ AND ‘substance use’** terms

**73 eligible papers included (after double screening)**

**Alcohol and Autism search string**

**Template for search strategy:**

**Alcohol use disorder terms based on:**

Conner, K. R., Pinquart, M., & Gamble, S. A. (2009). Meta-analysis of depression and substance use among individuals with alcohol use disorders. *Journal of substance abuse treatment*, *37*(2), 127-137.

Foulds, J., Newton‐Howes, G., Guy, N. H., Boden, J. M., & Mulder, R. T. (2017). Dimensional personality traits and alcohol treatment outcome: a systematic review and meta‐analysis. *Addiction*, *112*(8), 1345-1357.

Sileo, K. M., Miller, A. P., Wagman, J. A., & Kiene, S. M. (2021). Psychosocial interventions for reducing alcohol consumption in sub‐Saharan African settings: a systematic review and meta‐analysis. *Addiction*, *116*(3), 457-473.

Autism terms based on:

Do, B., Lynch, P., Macris, E. M., Smyth, B., Stavrinakis, S., Quinn, S., & Constable, P. A. (2017). Systematic review and meta‐analysis of the association of Autism Spectrum Disorder in visually or hearing impaired children. *Ophthalmic and Physiological Optics*, *37*(2), 212-224.

Hollocks, M. J., Lerh, J. W., Magiati, I., Meiser-Stedman, R., & Brugha, T. S. (2019). Anxiety and depression in adults with autism spectrum disorder: a systematic review and meta-analysis. *Psychological medicine*, *49*(4), 559-572.

Richards, C., Jones, C., Groves, L., Moss, J., & Oliver, C. (2015). Prevalence of autism spectrum disorder phenomenology in genetic disorders: a systematic review and meta-analysis. *The Lancet Psychiatry*, *2*(10), 909-916.

**Alcohol use disorder (OR):**

- Alcohol related disorders
- Alcohol addiction
- Alcohol abuse
- Alcohol dependence
- Binge drinking
- Alcoholi*
- Alcohol use
- Alcohol related disorders (MeSH)

**AND**

**Autism (OR):**

- Autis*
- Asperger*
- Pervasive developmental disorder
- ASD
- PDD
- Kanner
- Neurodevelopmental
- Child development disorders, pervasive (MesH)

**Search string:**

"Alcohol-Related Disorders"[Mesh] OR “alcohol use disorder”[Title/Abstract] OR “alcohol related disorders”[Title/Abstract] OR “alcohol addiction” [Title/Abstract] OR “alcohol abuse”[Title/Abstract] OR “alcohol dependence”[Title/Abstract] OR “binge drinking”[Title/Abstract] OR “alcoholi*”[Title/Abstract] OR “alcohol use”[Title/Abstract]

"Child Development Disorders, Pervasive"[Mesh] OR Autis*[Title/Abstract] OR Asperger*[Title/Abstract] OR “Pervasive developmental disorder” [Title/Abstract] OR ASD[Title/Abstract] OR PDD[Title/Abstract] OR Kanner[Title/Abstract] OR Neurodevelopmental[Title/Abstract]

**RESULTS**

198,366 hits for **alcohol terms**

94,241 hits for **autism terms**

694 hits for **‘autism’ AND ‘substance use’** terms

**26 eligible papers included (after double screening)**

**Appendix 3: Online Survey**

**What are your questions about substance, alcohol, and behavioural addictions in autism?**

Please watch one of our 3 minutes videos to learn more about our project and the survey.


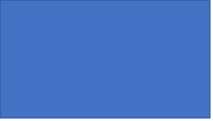

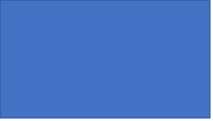


SABAA VIDEO SABAA VIDEO (SIMPLE VERSION)

Or alternatively for the written transcript, click [here](file:///C:/Users/ba1a21/Desktop/Survey%20explanation%20sheet.docx).

Please visit our website for more detailed information at <https://www.addiction-ssa.org/sabaa-substance-use-alcohol-and-behavioural-addictions-in-autism/>

For all our queries email us [sabaa@soton.ac.uk](mailto:sabaa@soton.ac.uk)

**Your consent**

By taking part in this survey, you consent to us storing and analysing the data you provide, so that the overall findings can be published. The findings will be published in a ‘pooled’ way: it will not be possible for data to be linked back to a particular person. Your answers will be anonymous. Any personal data will be stored and used in compliance with Data Protection Act 1998.

Every answer you give is valuable to us. Thank you very much for taking time to support us in this important project.

**Please tick the box below:***

- I give consent for my information to be used in this project and its publications. I am aware it will not be possible to link my answers back to me as an individual.

There are 23 questions in this survey. If you do not have any questions about a particular area, please leave that part blank. Questions that are marked with an asterisk (*) are required.

The survey will typically take anything between 5-30 minutes depending on your answers.

Click [here](https://teams.microsoft.com/l/file/2BE5BB34-121F-4394-A406-17A90C5DC902?tenantId=4a5378f9-29f4-4d3e-be89-669d03ada9d8&fileType=docx&objectUrl=https%3A%2F%2Fsotonac.sharepoint.com%2Fteams%2FSA%2FShared%20Documents%2FGeneral%2FSurvey%2FSurvey%20template%20280721.docx&baseUrl=https%3A%2F%2Fsotonac.sharepoint.com%2Fteams%2FSA&serviceName=teams&threadId=19:1f8cf144509e4293a390525eff87cddf@thread.tacv2&groupId=5db1d382-7444-4289-af8e-e71c17663888) to go to the survey.

1. **Problematic Alcohol use & Autism**
2. What do you think researchers should try to find out about **why** autistic people might drink too much alcohol in a way that harms them? Please list them below.
3. What questions do you have about **why** autistic people might drink too much alcohol in a way that harms them? Please list them below.
4. What do you think researchers should try to find out about how autistic people can be helped not to start drinking in a way that is harmful to them in the first place? Please list them below.
5. What questions do you have about how autistic people can be helped not to start drinking in a way that is harmful to them in the first place? Please list them below.
6. What do you think we should try to understand about how to find autistic people who are drinking too much, so they can be seen by someone who can help them? Please list them below.
7. What questions do you have about how can the health professionals find autistic people who are drinking too much, so they can be seen by someone who can help them? Please list them below.
8. What do you think researchers should try to find out how we can help autistic people drink less, if they are finding it hard to cut down or stop? Please list them below.
9. What questions do you have about how we can help people drink less, if they are finding it hard to cut down or stop? Please list them below.

1. What do you think it is important for researchers to find out about the best ways to support autistic people who are drinking too much, and to help them feel cared for? Please list them below.
2. What questions do you have about **caring and supporting** autistic individuals who use alcohol in a way that harms them? Please list them below.
3. What do you think researchers should try to find out about what challenges do the autistic people experience or what are the risk factors for them if they are drinking too much alcohol?
4. What questions do you have about **challenges and risks** for autistic individuals with problematic alcohol use? Please list them below.
5. Do you have **any other questions** that you think researchers should try to find out about autistic individuals who drink too much alcohol? Please list them below.

1. **Problematic Substance Use (Other than Alcohol) & Autism**
2. What do you think researchers should try to find out about **why** autistic people might use substances (other than alcohol) in a way that harms them? Please list them below.
3. What questions do you have about **why** autistic people might use substances (other than alcohol) in a way that harms them? Please list them below.
4. What do you think researchers should try to find out about how autistic people can be helped not to start using substances (other than alcohol) in a way that is harmful to them in the first place? Please list them below.
5. What questions do you have about how autistic people can be helped not to start using substances (other than alcohol) in a way that is harmful to them in the first place? Please list them below.
6. What do you think we should try to understand about how to find autistic people who are using substances (other than alcohol) in a way that harms them, so they can be seen by someone who can help them? Please list them below.
7. What questions do you have about how can the health professionals find autistic people who are using substances (other than alcohol) in a way that harms them, so they can be seen by someone who can help them? Please list them below.
8. What do you think researchers should try to find out how we can help autistic people to cut down substances that harms them, if they are finding it hard to cut down or stop? Please list them below.
9. What questions do you have about how we can help autistic people cut down on substances, if they are finding it hard to cut down or stop? Please list them below.
10. What do you think it is important for researchers to find out about the best ways to support autistic people who are using substances (other than alcohol) in a way that harms them, and to help them feel cared for? Please list them below.
11. What questions do you have about **caring and supporting** autistic individuals who use substances (other than alcohol) in a way that harms them? Please list them below.
12. What do you think researchers should try to find out about what challenges do the autistic people experience or what are the risk factors for them if they are using substances (other than alcohol) in a way that harms them alcohol?
13. What questions do you have about **challenges and risks** for autistic individuals who use substances (other than alcohol) in a way that harms them? Please list them below.
14. Do you have **any other questions** that you think researchers should try to find out about autistic individuals who use substances (other than alcohol) in a way that harms them? Please list them below.

1. **Behavioural Addictions^i^ & Autism**

*^i^Behavioural Addictions are repetitive behaviours that lead to problems for a person and/or their loved ones, such as gambling, gaming, etc.*

1. What do you think researchers should try to find out about **why** autistic people might have behavioural addictions that cause problems in their lives? Please list them below.
2. What questions do you have about **why** autistic people autistic people might have behavioural addictions that cause problems in their lives? Please list them below.
3. What do you think researchers should try to find out about how autistic people can be helped not to start having behavioural addictions that cause problems in their lives? Please list them below.
4. What questions do you have about how autistic people can be helped not to start having behavioural addictions that cause problems in their lives? Please list them below.
5. What do you think we should try to understand about how to find autistic people who have behavioural addictions that cause problems in their lives, so they can be seen by someone who can help them? Please list them below.
6. What questions do you have about how can the health professionals find autistic people who have behavioural addictions that cause problems in their lives, so they can be seen by someone who can help them? Please list them below.
7. What do you think researchers should try to find out how we can help autistic people quit their behavioural addictions, if they are finding it hard to stop? Please list them below.
8. What questions do you have about how we can help autistic people quit their behavioural addictions, if they are finding it hard to stop? Please list them below.
9. What do you think it is important for researchers to find out about the best ways to support autistic people who have behavioural addictions that cause problems in their lives, and to help them feel cared for? Please list them below.
10. What questions do you have about **caring and supporting** autistic individuals who have behavioural addictions that cause problems in their lives? Please list them below.
11. What do you think researchers should try to find out about what challenges do the autistic people experience or what are the risk factors for them if they have behavioural addictions that cause problems in their lives?
12. What questions do you have about **challenges and risks** for autistic individuals who have behavioural addictions that cause problems in their lives? Please list them below.
13. Do you have **any other questions** that you think researchers should try to find out about autistic individuals who have behavioural addictions? Please list them below.

1. **Populations in Autism**
2. Current research literature focuses on autistic people or people with autistic traits, individuals with self-diagnosis of autism, foetal alcohol spectrum disorder and pregnant women who use alcohol or substances during pregnancy. From your point of view, is there any other special population that researchers should focus on in the future?
3. Terminology
4. By problematic alcohol/substance use we mean the use of a substance that cause problems in people’s and their loved one’s lives. We acknowledge there is no universally accepted way to describe problematic use of alcohol or substances. From your perspective how would you define ‘problematic’ in this context? Please let us know and add your views below.

………………………………………………………………………………………………………………………………………………………………………………………………………………………………………………………………………………………………………………………..

It is important to us to hear from a wide range of people. To ensure that, we would like to ask a few questions to know more about you. Please choose the best option that describes you from the following lists.

1. **Which of the following describes you best? Please tick all that apply to you***

- A person who is autistic
- A person who has personal experience of substance, alcohol, or behavioural addiction (now or previously)
- A family member/carer/ friend for an autistic individual with substance, alcohol, or behavioural addiction (now or previously)
- A researcher, healthcare professional, professional or volunteer who works with autistic individuals
- A researcher, healthcare professional, professional or volunteer who works with people with addictions
- Other (please describe) …………………………….

1. **Further questions for autistic people***

**6.1.1. What is your link to autism? (tick any that apply)**

Diagnosed with one of the following:

- Autism
- Asperger’s syndrome or Asperger’s disorder
- Pervasive Developmental Disorder Not Otherwise Specified (PDD-NOS)
- Atypical autism
- Autism spectrum disorder/condition
- Self-diagnosed
- Never diagnosed but have a strong sense may be autistic
- Have autistic traits
- Other (Please specify) ……………………………………………

1. **What is your link to addictions?**

- A person who had/ has problematic substance use / addiction
- A person who had/ has alcohol use/alcohol addiction
- A person who has/ had behavioural addictions
- Other (Please specify) ……………………………………………

1. **Further questions for professionals***

**Are you (please tick all that apply to you)**

- Psychiatrist
- Researcher
- Psychologist
- Care worker
- Speech and language therapist
- Occupational therapist
- Psychotherapist
- GP
- Nurse
- Social worker
- Community worker
- Probation office
- Teacher
- Volunteer
- Other (please specify)………………..

1. **Where do you live? ***

- UK
- Europe
- USA
- Australia
- Middle East
- Asia
- Other Area (please specify) ..............................
- Prefer not to say

1. **Your ethnicity ***

- Please specify………………………………
- Prefer not to say

1. **What is your age in years? ***

- Please specify …………………………………
- Prefer not to say

1. **Do you identify as... ***

- Female
- Male
- Prefer to self-describe as: ………………………………
- Prefer not to say

1. **How did you find about this survey?***

- SSA website
- Other Website
- Twitter
- Email
- From family/friends
- Other (please specify) …………………………….

**If you have any comments/additional information, please write them below.**


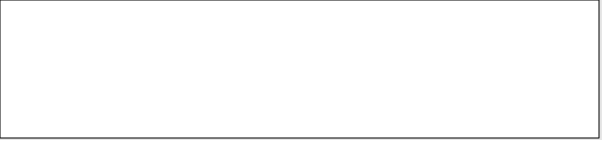


Thank you for your time and for completing this survey; your contribution is important to us. Please share this survey with anyone that you think might be interested.

**Would you like to join us for further steps?**

Would you like to hear from us for the next steps during the project? Or would you like to get involved in for the further steps including perhaps attending one of our stakeholder events? Please click on the link here ***[Link here] *** and complete the form.

Note that your questionnaire responses are anonymous – contacting us will not mean we can link answers back to you as an individual.

For further information, please refer to our website at

- <https://www.addiction-ssa.org/sabaa-substance-use-alcohol-and-behavioural-addictions-in-autism/>
- contact us on [sabaa@soton.ac.uk](mailto:sabaa@soton.ac.uk)
- Follow us on Twitter @PspSabaa

Appendix 4: Raw data

**ADDITIONAL INFORMATION ON DATA COLLECTION AND BACKGROUND DATA**

At the time of the study advertisement, two short videos were created (low sensory and animated versions) to accompany the survey to provide detailed information to the participants who may wish to take part^[[1]](#footnote-1)^. A transcript of the videos was also provided to enable enhanced accessibility. The survey was reviewed by the project team, and steering committee members and piloted by twenty-three people. The survey was open between Oct 11, 2021 and Jan 20, 2022.

The survey was advertised on the project website, social media accounts, various online newsletters and meetings and also by wider contacts and the steering group members ^[[2]](#footnote-2)^.

Raw survey data which is obtained through the survey was classified into themes using the NVivo software. These questions generated 38 ‘topic questions’ questions which formed the basis for the consensus excercise. The below table summarizes the information regarding the topic questions questions and their linked background raw data.^[[3]](#footnote-3)^

| Nu | Domain | Topic question | Background questions |
| --- | --- | --- | --- |
| 1 | Policy | What are the best ways to enhance awareness and acceptance of autism and reduce stigma; to better support autistic people to promote positive outcomes or prevent SABA? | 1. Can marketing be used as an effective tool to improve autism awareness and acceptance? 2. Instead of starting with the assumption of there is something wrong with autism that needs to be fixed, focusing on improving the communication between neurodiverse people and neurotypical people (wider society) might benefit the relationship. Would this be helpful in terms of reducing the stigma, increasing the connection, and therefore helping the prevention of addictions in autistic people? 3. What is the association between addiction, trauma, and autistic ableism? 4. Does the intersection of being an autistic person and a part of a marginalized group, affect these individuals in a negative way so that it results in the process of starting using substances, having behavioural addictions, or using alcohol in a problematic way? 5. Lack of autism awareness/understanding might end up with ineffective and even traumatizing therapy outcomes for autistic people. Can training medical professionals reduce the side effects of the therapies and increase the positive outcomes? 6. Is stigma about autism a factor in why autistic people start to drink? 7. Would learning how to manage the underlying triggers/causes of anxiety help autistic people not to start drinking in a way that is harmful to them in the first place? 8. Is there evidence that any of the following would help autistic people not to start drinking in a way that is harmful to them in the first place? 9. Provide young people with early intervention, educating them on what it is to be autistic/ neurodivergent? 10. Help them "find their tribe" and learn social skills? 11. build their self-esteem so they do not rely on alcohol and other substances to help them fit in? 12. Can raising awareness campaigns in primary care and community health teams help autistic people to seek for medical advice on their problematic alcohol use? 13. Can helping to build community connections help autistic people drink less or cut down alcohol? 14. What is the role of Education, awareness, key workers assigned, caught early before it progresses to further heavy use in the prevention of substances in autistic people? 15. Early help with coping skills and more acceptance in society of the differences. Education on the harms of alcohol and de-glamorising of it in general. 16. What could be done if the person does not think they have a problem? 17. Can learning about autism help autistic individuals to maintain a good recovery from behavioural addictions? |
| 2 | Policy | What are the current barriers and enablers in healthcare services for autistic people (including those from BAME backgrounds) seeking help for SABA? | 1. Which barriers from the list below exemplify the barriers that BAME communities experience in mainstream services?  - Shame, stigma and discrimination - Lack of culturally appropriate support - Lack of choice - Lack of consideration given to faith and spirituality-based recovery options - Lack of recovery programs tailoring to cultural specificity, beliefs, and values - Language barriers - People attempting to handle problems themselves, the shame of asking for help and mistrust of services  1. What impact does the environment (of the healthcare services) have on engaging autistic people? Do service settings and environments present barriers to autistic people accessing help? 2. How can we best adapt the environment, for example, to clinical services so that they're very more welcoming to people to autistic people and suitable to their needs? 3. How can we be more responsive to autistic individuals who access support where support is unlikely to be offered in their first language? 4. How do we ensure services are as responsive as possible to the breadth of autistic communication needs? 5. Trauma-informed approach is not currently well implemented in NHS services, what impact does this have on autistic people developing addictions? 6. What are the barriers autistic people face in mental health services that worsen their symptoms/add on their current difficulties? 7. What are the challenges that autistic people from BAME background face when they experience addictions? 8. In the absence of effective services, family/ greater family becomes the primary source of support for autistic people from BAME backgrounds who have addictions. What are the experiences of those people during these challenging times, what are the possible challenges? 9. Does alcohol use become problematic for autistic people because current alcohol recovery units working hours has time limitations that do not fit with some autistic individuals’ strict time arrangements caused by rigidity in thinking, therefore they cannot seek help when needed? 10. would more mental health support, more community services, faster diagnosis and shorter waiting times for diagnosis help autistic people not to start drinking in a way that is harmful to them in the first place? 11. How easy is it for autistic people to access skills training or mental health support centres? And what are the main barriers for autistic people to access these centres? 12. What are autistic individuals’ perceptions and needs about the support and care that they have? 13. Are the current treatments tailored to suit autistic individuals’ neurological needs? 14. How do current practices deal with the autism stigma when working with autistic individuals who have problematic alcohol use? 15. How can physical conditions of substance use treatment offices be adapted to suit autistic individuals' needs? 16. Are there any examples of autism-adapted practice that we can apply to addiction services? 17. Can mainstream specialist gambling services support people with autism? What adaptations are required? Or do we need specialist services to in-reach into autism services |
| 3 | Policy | What are the training needs of families/carers or other professionals (e.g. teachers) working with autistic people with SABA? | 1. Families’ and society’s understanding of autism and addictions, and the stigma around these conditions might have an impact on individuals’ mental health. How do these two conditions affect autistic people’s developing addictions? 2. What support or educational tools would be helpful so that carers/friends/family of people with autism can help identify a problem and encourage the person with autism to seek help? 3. Would social workers or care home workers help identifying autistic people who need help with their problematic alcohol drinking? 4. What is the role of Education, awareness, key workers assigned, caught early before it progresses to further heavy use in the prevention of substances in autistic people? 5. Would educating carers be helpful in the prevention of substance use in autistic people? 6. Does educating parents/ carers help preventing behavioural addictions in autistic people? 7. What structures and schedules can carers put in place to limit the risk of these addictions? what structural or environmental risks can be removed to create a healthy, low-risk environment? |
| 4 | Policy | What training do professionals in different sectors need to enable them to better support autistic people who have SABA? | 1. Can preventative early intervention works in schools, colleagues, universities, or specified target businesses with early intervention support (as high functioning autism can result in individuals being in such roles) help finding autistic people with problematic alcohol use so that they could be seen by a health professional? 2. How can professionals successfully reach out to people with autism so they can be aware of problematic alcohol use and how to seek help? 3. Do health professionals have a good understanding of neurodiversity and autism? 4. Would social workers or care home workers help identify autistic people who need help with their problematic alcohol drinking? 5. Can improving professional awareness around ASD teens who may be more vulnerable to substance misuse help improving the quality of targeted early interventions? |
| 5 | Policy | What adaptations to screening/ diagnostic tools are needed for more accurate and precise diagnoses of SABA in autistic people? | 1. Are there any specific diagnostic tools used to detect problematic alcohol use of autistic individuals? 2. Do autism health checks include alcohol use-related questions to detect and refer autistic individuals where needed? 3. Are the screening procedures in current practices autism friendly so that autistic people could be open to sharing their drinking-related problems? 4. Would identifying people who have problematic alcohol use and screening them for autism and other neurodivergencies help us to find autistic individuals who have problematic alcohol use so that they could be seen by someone who can help them? 5. What are the early warning signs of autistic people who have problematic alcohol use? Are there any special screening tests that can be applied to autistic people in terms of problematic alcohol use? 6. How to communicate more effectively about addiction (e.g. non shaming language) and tools in addiction recovery? 7. Is regular screening a helpful tool to detect substance use in autistic people? 8. Is regular screening by GP or nurses a helpful tool to detect substance use in autistic people? 9. Amongst people who are accessing services, would we be able to screen for autism if it is not a known diagnosis already? 10. How do the screening procedures work for autistic people, is there any ways of improving them? 11. Can defining the distinction between 'typical' and 'problematic/ inappropriate' help us in the prevention of behavioural addictions in autistic people? 12. Does the uncertainties about behavioural addictions area limit diagnosis options of behavioural addictions in autistic people? 13. What adaptions can be done in current practices to diagnose autism and behavioural addictions more precisely/accurately so that people affected by these conditions could benefit from treatment? |
| 6 | Policy | Would providing healthy alternatives to meet autistic individuals’ needs help to prevent autistic people developing SABA? | 1. Is there a healthy alternative to meet autistic individuals’ needs that they hope to meet by drinking alcohol? 2. would preventative support around healthier coping mechanisms for difficulties such as social events, and coping with breaking compulsions help autistic people not to start drinking in a way that is harmful to them in the first place? 3. Can providing alcohol-free socialising places help reduce problematic alcohol use? 4. Would it help us to care for and support autistic individuals better by taking into account the needs of autistic individuals when re-evaluating conditions of care homes e.g., allowing drinking in care homes to prevent binge drinking in other places such as pubs. 5. Can providing alternatives to the current model of socialising which benefits extroverts and drinkers help autistic people to drink less 6. Do they have enough access to other support mechanisms to prevent them from relying on alcohol? 7. Does supporting the development of heathy coping mechanisms help autistic individuals not to start using substances (other than alcohol) in the first place? 8. What alternative/ non harmful self-soothing/repetitive behaviours can take the place of behavioural addictions? what are the mutable risk factors that can be targeted to prevent behavioural addictions? |
| 7 | Policy | Would screening for SABA in autism be an effective form of prevention? | 1. Would mental health professionals or GPs help autistic people to get help with their problematic alcohol use with regular screening? 2. What threshold do autistic people think they are drinking to excess 3. Do services supporting autistic people routinely screen for addictions? 4. What standard questions can be asked in services to ID substance misuse? 5. Can regular screening/ early diagnosis help reduce the harm caused by behavioural addictions? 6. Is gambling behaviour included in general health and wellbeing checks so that autistic individuals can benefit from that as well? 7. what are the screening tools/questions that can be asked to identify people at risk for behavioural addictions? 8. Is regular screening by GP or nurses a helpful tool to detect behavioural addictions in autistic people? |
| 8 | Policy | Would employing professionals specialized in both autism and SABA be more effective in promoting positive outcomes in autistic people with SABA? | 1. Lack of autism awareness/understanding might end up with ineffective and even traumatizing therapy outcomes for autistic people. Can training medical professionals reduce the side effects of the therapies and increase the positive outcomes? 2. And would autism charities be prepared to fund places specialist units. 3. Do uncertainties about behavioural addictions area limit the diagnosis of behavioural addictions in autistic people? 4. Can mainstream specialist gambling services support people with autism? What adaptations are required? Or do we need specialist services to in-reach into autism services |
| 9 | Policy | What is the best way of applying person-centred care in SABA services so that all people can benefit (including autistic people and people from minority groups e.g. gender-diverse people)? | 1. Autistic people could be treated differently by the healthcare staff if their diagnosis is known to them, Can anonymity might help? 2. How can we best adapt the environment, for example, the clinical services and so that they're very more welcoming to people to autistic people and suitable to their needs? 3. How can we be more responsive to autistic individuals who access support where support is unlikely to be offered in their first language? 4. In the absence of effective services, family/ greater family becomes the primary source of support for autistic people from BAME backgrounds who have addictions. What are the experiences of those people during these challenging times, what are the possible challenges? 5. What adaptations could be made to inpatient services to make them accessible and more suitable to autistic peoples’ needs? 6. When adapting existing therapies/ developing novel ones can person-centred care which acknowledges individual differences help prevent/treat addictions in autistic people? 7. Is the ratio of problematic alcohol use differs in different cultures, especially in cultures where alcohol is commonly used in social situations and the contrary. 8. Does alcohol use become problematic for autistic people because current alcohol recovery units working hours has time limitations that do not fit with some autistic individuals’ strict time arrangements caused by rigidity in thinking, therefore they cannot seek help when needed? 9. Are there any differences between problematic alcohol use treatment entry and utilization in autistic individuals with and without intellectual disabilities and the general population? 10. Would not limiting the target patient group to autistic people- keeping it broad to include underdiagnosed autistic individuals help reduce the prevalence? 11. What arrangements can be done for a more autism-friendly alcohol recovery? And what autistic features should be taken into account when making these arrangements (such as rigidity, social difficulties, repetitive behaviours etc.)? Can any of those be taken as strengths when designing interventions? 12. Are the current treatments tailored to suit autistic individuals’ neurological needs? 13. Is it possible to meet the specific needs of people living with autism and also promote integration with others? 14. How do treatment entry and utilization compare for individuals with intellectual disabilities and the general population? 15. How can physical conditions of substance use treatment offices be adapted to suit autistic individuals' needs? 16. Are there any examples of autism-adapted practice that we can apply to addiction services? 17. what are autistic individuals’ thoughts/perceptions on the support/care they have accessed for? 18. Can mainstream specialist gambling services support people with autism? What adaptations are required? Or do we need specialist services to in-reach into autism services? |
| 10 | Policy | What are the most effective ways of displaying information about legislation; guidance and education (e.g. in harm reduction campaigns; educational materials; treatment material etc.) to facilitate engagement of autistic people in prevention; treatment; care or support of SABA? | 1. Can marketing be used as an effective tool to improve autism awareness and acceptance? 2. What is the best way of giving information to autistic people about alternative coping strategies? 3. How is information best displayed to autistic people (low sensory harm reduction campaigns etc)? 4. What impact does current packaging and campaigns (e.g. 'childlike', bright coloured IPA packaging etc.) have on autistic people? 5. Would providing clear guidelines about when and who to contact help autistic people seek advice in time? 6. Are there any informative booklets, call centers, or web pages in terms of problematic alcohol use in autistic people for their relatives? 7. Which kind of communication would help when prevention of substance use is aimed in autistic people? 8. Are current guidelines clear enough for autistic people/autism friendly? 9. how can interventions be delivered in formats that are acceptable to people with autism- including both verbal and nonverbal individuals 10. what education, skills and support autistic people might benefit from in early adolescence 11. Can public advertising help finding autistic people who have behavioural addictions, so they can be seen by someone who can help them |
| 11 | Policy | What do professionals need to better care for autistic people with SABA? | 1. Autistic people could be treated differently by the healthcare staff if their diagnosis is known to them, can anonymity might help? 2. Lack of autism awareness/understanding might end up with ineffective and even traumatizing therapy outcomes for autistic people. Can training medical professionals reduce the side effects of the therapies and increase the positive outcomes? 3. How can healthcare professionals be helped to accurately identify conditions with overlapping symptoms (which can be hard to disentangle) such as ADHD and ASD? Can one/both of those diagnoses impact autistic people negatively acting as a risk factor to develop addictions? 4. What are the key skills that a healthcare professional would need when working with autistic people who have addictions? 5. Are GPs aware of the risks of problematic alcohol use in autistic individuals? 6. Would raising awareness of GPs help to find and refer autistic individuals who need treatment for problematic alcohol use? 7. How can professionals successfully reach out to people with autism so they can be aware of problematic alcohol use and how to seek help? 8. Do health professionals have a good understanding of neurodiversity and autism? 9. Would it be helpful if GPs/school counselling/CAMHs were trained not to consider early issues as 'normal teenage development’? 10. Are the current treatments tailored to suit autistic individuals’ neurological needs? 11. What training do recovery staff have around autism? What impact does autism have (if any) on routine (such as where medication is used to treat addiction. 12. What is the autism-related training needs of substance recovery workers? 13. What is the treatment needs of the professionals who work with autistic individuals who have addictions? 14. Can defining the distinction between 'typical' and 'problematic/ inappropriate' help us in the prevention of behavioural addictions in autistic people? |
| 12 | Policy | How can we introduce language to move society from stigma to celebration in order to reduce the negative effects of stigma on autistic people with SABA? | 1. Which barriers from the list below exemplify the barriers that BAME communities experience in mainstream services?   • Shame, stigma and discrimination  • Lack of culturally appropriate support  • Lack of choice  • Lack of consideration given to faith and spirituality-based recovery options  • Lack of recovery programs tailoring to cultural specificity, beliefs, and values  • Language barriers  • People attempting to handle problems themselves, the shame of asking for help and mistrust of services   1. Can marketing be used as an effective tool to improve autism awareness and acceptance? 2. Instead of starting with the assumption of there is something wrong with autism that needs to be fixed, focusing on improving the communication between neurodiverse people and neurotypical people (wider society) might benefit to the relationship. Would this be helpful in terms of reducing the stigma, increasing the connection and therefore help the prevention of addictions in autistic people? 3. What is the association between addiction, trauma and autistic ableism? 4. Is stigma about autism a factor in why autistic people start to drink? 5. Is there evidence that any of the following would help autistic people not to start drinking in a way that is harmful to them in the first place? 6. support to understand the risks like anyone else but with reference to the specific social difficulties they face? 7. Explanations that alcohol is a false friend for them? 8. Help in early childhood and adolescence to manage emotions? 9. Emotional coping skills and healthy ways to manage mood and understand their differences in a non-judgemental way? 10. Society as a whole to not stigmatise those with autism and accept their presence in the rich social fabric so they don't feel under pressure 11. How does autistic peoples’ perceptions of healthcare staff and wider society affect their openness to seek advice? 12. Can providing alcohol-free socialising places help reduce problematic alcohol use? 13. Can helping to build community connection help autistic people drink less or cut down alcohol? 14. How to communicate more effectively about addiction (e.g., non shaming language) and tools in addiction recovery? 15. Do autistic people feel discriminated against by society? 16. How do current practices deal with the autism stigma when working with autistic individuals who have problematic alcohol use? 17. How do we find a way to allow those with ASD to feel connected in a world which is largely difficult to navigate socially? 18. Are Empowerment within their communities, less stigma, better employment and education support helpful in preventing substance use in autistic people? 19. Early help with coping skills and more acceptance in society of the differences. Education on the harms of alcohol and de-glamorising of it in general. 20. What impact does the stigma against drug use and related criminal queries have on autistic people open up themselves if they use substances? 21. How can we remove stigma of a gambling disorder? 22. Is the difficulty of trying any of these contribute to behavioural addictions in autistic people; trying to feel OK, connect socially and deal with sensory and emotional dysregulation? |
| 13 | Practice | What adaptations to current approaches are needed to increase effectiveness of SABA treatment for autistic people? | 1. What are the alternatives to only-Western-based therapies, an enhanced trauma care approach that also includes consequences of recurrent racism and ableism?’ 2. What are the alternative delivery formats such as non-face-to-face, digital, alternative media/sensory inputs?’ 3. Can improvements to current trauma care practice, by including culturally informed trauma help, support addiction treatment in autistic people? 4. Can adopting a holistic approach rather than just focusing on separate diagnoses help health professionals better define the ‘root problems’ in autistic people’s lives in a better way and therefore enable health professionals to treat the core problems more effectively? 5. Also, does it help measuring the side effects of the medications and therapy for the particular person? 6. What are the potential therapies that we could re-purpose to be used in autistic people with addictions? 7. When adapting existing therapies/ developing novel ones can person-centred care which acknowledges individual differences help to prevent/ treat addictions in autistic people? 8. How can the environmental conditions best adapt to autistic needs for example considering the difficulties that group therapies might bring, individual therapies might be more useful when working with autistic people on their addictions. 9. Given that most of the therapies are western based, how can we increase the options open to autistic people who need help with their addiction-related problems? 10. What type of psychoeducation is needed? 11. How can current practices be adapted to address autistic individuals’ needs who drink alcohol in a problematic way? 12. How Motivational Interviewing can be adapted for autistic people who have a problematic alcohol use? 13. How can contingency management intervention be adapted to be suitable for individuals with autism? 14. Are there modified alcohol reduction programmes for those with ASD? 15. Is using tools more effective compared to involving 'human interaction' when helping autistic people in their alcohol recovery process? 16. What are the drawbacks of current therapies that are aiming to reduce alcohol consumption in autistic individuals? 17. What arrangements can be done for a more autism-friendly alcohol recovery? And what autistic features should be taken into account when making these arrangements (such as rigidity, social difficulties, repetitive behaviours etc.)? Can any of those be taken as strengths when designing interventions? 18. What alternative delivery formats (e.g., non face to face, digital, alternative media/sensory inputs) can be harnessed for effective delivery. 19. Are there any examples of autism-adapted practice that we can apply to addiction services? 20. Can focusing on reducing harm rather than reducing intake a more realistic target for autistic people who use substances? 21. What adaptations can be done in treatment to make it respond better to autistic individuals' needs? 22. How can we promote treatment maintenance after someone is discharged or following treatment completion? 23. What adaptations can be done in treatment to make it respond better to autistic individuals' needs? |
| 14 | Practice | Do autistic and non-autistic people differ in the factors involved in SABA vulnerability chronicity; and response to treatment? | 1. Are autistic people more or less likely to binge drink? 2. Are they likely to have atypical reactions to alcohol, as they sometimes do to medication so they experience it differently? 3. Why are some people more vulnerable to developing it than others? 4. Do the same treatments work for people with autism compared to people without autism? 5. Is there a greater conversion rate from substance use to substance use disorder in autistic people compared to the general population? 6. Are autistic people differ from their neurotypical peers in terms of age, age of onset, socioeconomic status, etc when starting to use alcohol in a problematic way? 7. Are autistic people more likely to suffer an addiction which contributes to their problematic alcohol use? 8. Are the current treatments tailored to suit autistic individuals’ neurological needs? 9. Compared to their neurotypical peers do autistic people have different maintaining factors to addition and how can we break them? 10. Is there a difference between those who use substances other than alcohol? are there sensory factors involved? if they use one type of substance is it progressive or do they tend to stick to one? 11. Do narcotics affect autistic people differently? 12. Why are autistic people more likely to become addicted? 13. Considering the research showing differences in the effects of psychedelics in this group, are there differences in the way that substances affect autistic people? Would be helpful to know about stimulants etc as this may then inform the discussion about why people get into problem substance use. 14. Are causal factors similar to those seen in non-autistic groups 15. Are behavioural addictions more common in people with autism vs not? 16. Do they present the same way? 17. Do they develop over time in the same way? 18. What safeguards would be helpful to prevent people with autism developing problem gambling? are these in addition to, or similar to, those for people without autism? 19. What harms are associated with gambling disorder and people with autism? |
| 15 | Practice | In terms of treatment and promoting positive outcomes; what are the most helpful elements or approaches in the management of SABA in autistic people? | 1. What are the potential benefits of peer support and mentoring during recovery? 2. Can medical cannabis help treating addictions in autistic people by reducing the anxiety symptoms which might be a triggering factor for developing addictions? 3. Can emotion regulation therapies/ psychoeducation programmes help prevent addictions in autistic people by reducing their anxiety caused by difficulties identifying and regulating their emotions? 4. Can enhancing autistic individuals’ capacity to deal with ableism or racism help them to cope with life challenges and therefore prevent/ combat addictions? 5. Are AA and 12-step programmes catering effectively to autistic needs? 6. Is it effective to try and get them to regulate the amount they drink by the number of drinks they have had rather than how they are feeling? 7. How trauma therapy would help as an intervention even for people who do and do not identify a single traumatic event. How yoga geared towards trauma recovery would help as an intervention. 8. do usual treatment approaches (SBIRT, 1:1 counselling, group work) work as well, better or worse for autistic people. 9. Any strategies that are particularly effective for autistic people? 10. Do any of the following work effectively for autistic individuals to drink less, if they are finding it hard to cut down or stop? 'drink diaries, online support forums, support groups, structured alcohol groups, enhancing support networks, secure and safe housing, coping strategies, ITEP maps, Decision balance' 11. Does 'a quick question' technique work effectively for autistic individuals drink less, if they are finding it hard to cut down or stop? 12. Is mental health support provided in a timely manner effective to help autistic individuals to drink less? 13. Do guiding autistic individuals by providing them clear guidelines about how to measure their alcohol and encouraging them to keep accurate records help autistic individuals to drink less? 14. Do emotional coping skills help them to reduce alcohol intake? 15. Can brief interventions or ASSIST tools/approaches help autistic individuals to drink less/cut down or stop? 16. What programmes work, such as ACTs, CBT, pharmacological treatments etc? 17. What types of interventions actually work for autistic people? Do interventions that focus on particularly dealing with the social issues help more than treatments focusing on alcohol itself? 18. Can helping to build community connections help autistic people drink less or cut down on alcohol? 19. what interventions might be useful for caring for and supporting autistic individuals? 20. Can motivational Interviewing techniques be used to raise awareness as a part of care and support? 21. Does the care plan approach work good for people who are willing to solve their problematic alcohol use problem? 22. Can reducing anxiety that autistic individuals experience supports autistic individuals in their alcohol recovery process? 23. What is the best modality of PSI in autistic individuals with AUD/SUD? 24. Do autistic individuals with problematic alcohol use respond better to one-to-one therapies compared to group therapies? 25. Can being in contact with autistic people who had recovered/are in recovery from problematic alcohol use be helpful for autistic individuals who would like to reduce their alcohol intake? 26. Are there any interventions that can help carers support reductions in problem drinking among autistic individuals? 27. Can supporting the carer to be more resilient/ less vulnerable to burnout can help the process of curing problematic alcohol use? 28. Would interventions targeting both autistic individuals and their carers be more helpful in alcohol recovery? 29. How can we promote treatment maintenance after someone is discharged or following treatment completion? 30. Can any of the following might be helpful in reducing/cutting down substance use in autistic individuals; Education on units, weekly meetings with a therapist, looking at dual diagnosis and the local community mental health team involvement 31. Can support programs help reduce substance use? 32. Are Community Reinforcement or Family Therapy effective techniques for autistic individuals who use substances? 33. Can training programs on identifying overlaps in autism and behavioural addiction behaviours be helpful when treating behavioural addictions? 34. Is there any evidence that shows any of the following factors help autistic individuals to recover from behavioural addictions?  - Weekly counselling meetings, - AA zoom meetings, - Anti-craving meds, - CBT etc.  1. Is CBT an effective treatment in autistic individuals with behavioural addictions? 2. what interventions work to reduce these repetitive behaviours and to what extent are these feasible to implement and designed for people with autism? |
| 16 | Practice | What are the early indicators for developing SABA in autistic people and how can autistic people and their families identify these indicators? | 1. What 'early signals' indicate drinking is becoming a problem in someone with autism? 2. Would it be helpful if GPs/school counselling/CAMHs were trained not to consider early issues as 'normal teenage development'? 3. How can autistic people realise they have a problem with alcohol and how to help them communicate about it to the right person that can direct to help? 4. What are the early warning signs of autistic people who have problematic alcohol use? Are there any special screening tests that can be applied to autistic people in terms of problematic alcohol use? 5. Are there other strategies beyond the usual screening processes that can be used to identify people at risk for problem drinking? 6. Is regular screening by GP or nurses a helpful tool to detect substance use in autistic people? |
| 17 | Practice | How can families be supported to limit risks; promote prevention of SABA and promote positive outcomes for autistic people? | 1. When there is a family member using substances or alcohol, what is the impact on the other family members, siblings, the psychological and emotional impact? 2. Can preventative early intervention works in schools, colleagues, universities, or specified target businesses with early intervention support (as high functioning autism can result in individuals being in such roles) help finding autistic people with problematic alcohol use so that they could be seen by a health professional? 3. How to organise ambulatorial assistance to support families without isolating the individuals in need of help? 4. Are there any interventions that can help carers support reductions in problem drinking among autistic individuals? 5. Can supporting the carer to be more resilient/ less vulnerable to burnout can help the process of curing problematic alcohol use? 6. Would interventions targeting both autistic individuals and their carers be more helpful in alcohol recovery? 7. Would educating carers be helpful in the prevention of substance use in autistic people? 8. what kinds of information and education materials and coping strategies would benefit carers? 9. what education, skills and support autistic people might benefit from in early adolescence 10. What support is available for people/carers affected by a person with autism and a gambling disorder? |
| 18 | Practice | What is the best way of supporting children who are living with parents who are autistic and have SABA? | 1. When there is a family member using substances or alcohol, what is the impact on the other family members, and siblings, the psychological and emotional impact? 2. 'What is the best way of supporting children who are living with parents who are autistic and have problematic alcohol use? ' 3. How high is the risk of suicide and do they often feel themselves as a burden to their families? 4. Considering problematic alcohol use during the perinatal period and its outcomes in offspring (autistic traits in babies), is the mum’s drinking related to her autism, and is a baby autistic because it's genetic and coming from the mum? |
| 19 | Practice | Is unrecognised autism (ie not diagnosed and/or self-identified) a risk factor for developing SABA? | 1. Is the age of diagnosis/recognition or identification of autism a predictor factor of SABA in later life? If autism isn't diagnosed early, if it's a late diagnosis, might that more likely predict SABAA in later life? 2. If the indicators of behavioural addictions are missed in individuals (late diagnosis) and/or autism is underdiagnosed how do this affect treatment outcomes? 3. Whether or not the group of undiagnosed autistic people (many of whom may be women) may be at real risk for developing addictions. Might they be a particularly vulnerable group for substance misuse? 4. Is there any association between late diagnosis and problematic alcohol drinking? 5. Would not limiting the target patient group to autistic people- keeping it broad to include underdiagnosed autistic individuals help reducing the prevalence? 6. Does age at diagnosis (and masking) correlate with the number of autistic individuals with problematic alcohol use? 7. Do children who are diagnosed with autism receive enough post-diagnostic support at the point of diagnosis? If they did would this support them to get a better understanding of themselves and autism and be enough to prevent alcohol misuse? 8. Is being undiagnosed a risk factor for autistic people in starting to use substances as a coping mechanism maybe, particularly in teenage or young adulthood (feeling and acting different from their peers but not knowing the reasons- trying to be a part of a group)? 9. When were these diagnosed? How did their diagnosis affect their substance use? How did the timing of their autism diagnosis affect their substance use? |
| 20 | Practice | What are the characteristics in autism that could be beneficial in positive outcomes from SABA? | 1. Can some autistic features such as 'following rules' considered as strengths when aiming to reduce alcohol intake? 2. What arrangements can be done for a more autism-friendly alcohol recovery? And what autistic features should be taken into account when making these arrangements (such as rigidity, social difficulties, repetitive behaviours etc.)? Can any of those be taken as strengths when designing interventions? 3. Can a better understanding of ASD and its effects on substance use be beneficial for the prevention of substance use? 4. Can focusing on neurology and looking at the strengths that hyper-focus brings and challenge autistic peoples' energy into something productive? |
| 21 | Practice | Could improving all services that support autistic people help to prevent SABA and promote positive outcomes in autistic people? | 1. Autistic people could be treated differently by the healthcare staff if their diagnosis is known to them, can anonymity might help? 2. How can we best adapt the environment, for example, to clinical services so that they're very more welcoming to people to autistic people and suitable to their needs? 3. Can improvements to current trauma care practice, by including culturally informed trauma help, support addiction treatment in autistic people? 4. How do we ensure services are as responsive as possible to the breadth of autistic communication needs? 5. Trauma-informed approach is not currently well implemented in NHS services, what impact does this have on autistic people developing addictions? 6. What adaptations could be made to inpatient services to make them accessible and more suitable to autistic peoples’ needs? 7. Considering current PTSD treatments target past traumas, not existing ones, what is the best way to help autistic people who have addictions when they are suffering from the effects of trauma caused by recurrent racism and ableism? 8. Can autistic people be effectively treated in neurotypical early intervention services? 9. Would providing clear guidelines about when and who to contact help autistic people seek advice in time? 10. Are the current treatments tailored to suit autistic individuals’ neurological needs? 11. Are there specialist rehab facilities? 12. What kind of adaptations would be helpful in alcohol and drug services to make them more effective for autistic individuals? |
| 22 | Practice | Are there specific characteristics in autism which may be protective against developing SABA? | 1. What are the coping strategies that could help autistic people to feel less social anxiety in social situations so that they could replace alcohol with those? 2. Look at the opposite- what is the difference between autistic people that do not rely on alcohol and substances? What are the protective factors- a guess is that they have channelled their energy into other areas that provide healthy outcomes? They are probably surrounded by people that understand them - family, education and employment (this is rare). Also, the role that self-esteem plays in addiction. |
| 23 | Practice | What are the most effective ways to prevent SABA in autistic people (at legislation; policy and practice levels)? | 1. Can enhancing autistic individuals’ capacity to deal with ableism or racism help them to cope with life challenges therefore prevent/ combat addictions? 2. would preventative support around healthier coping mechanisms for difficulties such as social events, coping with breaking compulsions help autistic people not to start drinking in a way that is harmful to them in the first place? 3. would more mental health support, more community services, faster diagnosis and shorter waiting times for diagnosis help autistic people not to start drinking in a way that is harmful to them in the first place? 4. Would learning how to manage the underlying triggers/causes of anxiety help autistic people not to start drinking in a way that is harmful to them in the first place? 5. Is there evidence that any of the following would help autistic people not to start drinking in a way that is harmful to them in the first place?  - support to understand the risks like anyone else but with reference to the specific social difficulties they face. - Explanations that alcohol is a false friend for them? - Help in early childhood and adolescence to manage emotions? - Emotional coping skills and healthy ways to manage mood and understand their differences in a non-judgemental way? - Society as a whole to not stigmatise those with autism and accept their presence in the rich social fabric so they don't feel under pressure  1. What practical strategies can be developed to help stop problematic alcohol use developing in autistic people? Are these the same or different from those helpful for non-autistic people? 2. Is there evidence that any of the following would help autistic people not to start drinking in a way that is harmful to them in the first place?  - Provide young people with early intervention, educating them on what it is to be autistic/ neurodivergent? - Help them "find their tribe" and learn social skills? - Build their self-esteem so they do not rely on alcohol and other substances to help them fit in?  1. Would understanding the impact on the health of drinking over a certain number of units and gaining a better understanding of their social interaction difficulties help autistic people not to start drinking in a way that is harmful to them in the first place 2. What are the coping strategies that could help autistic people to feel less social anxiety in social situations so that they could replace alcohol with those? 3. Do strengthening emotion regulation or coping skills, transdiognostically, help autistic people become more resilient to life challenges? 4. Can preventative early intervention works in schools, colleagues, universities, or specified target businesses with early intervention support (as high functioning autism can result in individuals being in such roles) help finding autistic people with problematic alcohol use so that they could be seen by a health professional? 5. Can providing alcohol-free socialising places help reduce problematic alcohol use? 6. Do they have enough access to other support mechanisms to prevent them from relying on alcohol? 7. Do children who are diagnosed with autism receive enough post diagnostic support at the point of diagnosis? If they did would this support them to get a better understanding of themselves and autism and be enough to prevent alcohol misuse? 8. From a prevention point of view, is it helpful discussing the pros and cons of using substances or making arrangements to help autistic people to be involved in new activities/people that might be interest of them? 9. Which kind of communication would help when prevention of substance use is aimed in autistic people? 10. Does supporting the development of heathy coping mechanisms help autistic individuals not to start using substances (other than alcohol) in the first place? 11. What is the role of Education, awareness, key workers assigned, caught early before it progresses to further heavy use in prevention of substances in autistic people? 12. Are Empowerment within their communities, less stigma, better employment and education support helpful in preventing substance use in autistic people? 13. what preventative PSI might be helpful 14. Early help with coping skills and more acceptance in society of the differences. Education on the harms of alcohol and de-glamorising of it in general. 15. Can a better understanding of ASD and its effects on substance use be beneficial for prevention of substance use? 16. What are autistic individuals’ opinions on helpful preventative approaches? 17. How can we safeguard autistic individuals from being forced or tricked into using substances? How do we prevent substance use among autistic individuals during childhood (under the age of 12 years) 18. What could be done to stop substance use become a pattern in the first place? 19. Are training schemes effective tools to prevent substance use in autistic individuals? 20. What could be done to prevent autistic people to develop gaming addiction if socialising is a part of the gaming problem? 21. what education, skills and support autistic people might benefit from in early adolescence 22. Can defining the distinction between 'typical' and 'problematic/ inappropriate' help us in prevention of behavioural addictions in autistic people? 23. What safeguards would be helpful to prevent people with autism from developing problem gambling? are these in addition to, or similar to, those for people without autism? 24. Can public advertising help finding autistic people who have behavioural addictions, so they can be seen by someone who can help them 25. Can training programs on identifying overlaps in autism and behavioural addiction behaviours be helpful when treating behavioural addictions? |
| 24 | Practice | If early indicators of developing SABA were overlooked in autistic individuals how would this affect treatment outcomes? | 1. Is the age of diagnosis/recognition or identification of autism a predictor factor of SABA in later life? If autism isn't diagnosed early, if it's a late diagnosis, might that more likely predict SABAA in later life? 2. If the indicators of behavioural addictions are missed in individuals (late diagnosis) and/or autism is underdiagnosed how do this affect treatment outcomes? 3. What is the impact of early/late diagnosis of autism on seeking treatment? Are health professionals trained to detect the first steps of autism? 4. Would it be helpful if GPs/school counselling/CAMHs were trained not to consider early issues as 'normal teenage development'? 5. Are behavioural addictions missed in autistic people because they are assumed to be a part of autism phenotype? 6. Can regular screening/ early diagnosis help reduce the harm caused by behavioural addictions? |
| 25 | Practice | Do autistic individuals respond differently to current legislation/regulations around SABA? | 1. Do autistic people tend to follow the rules, e.g., do not buy alcohol if they are under 18 or do they think they are able to make their own decisions about their alcohol consumption and do not need someone else to make a law about it? 2. Do autistic individuals respond differently to current legislation/regulations around SABA? 3. Does curiosity towards substances lead perhaps to develop an addiction later in life? Is it related to sensory-seeking behaviour, following other people saying it is a good or interesting sensory experience? |
| 26 | Practice | Is the risk of developing SABA increased in autistic women due to late diagnosis of autism? | 1. Is the risk of developing SABA increased in autistic women due to late diagnosis of autism? 2. Whether or not the group of undiagnosed autistic people (many of whom may be women) may be at real risk for developing addictions? Might they be a particularly vulnerable group for substance misuse? |
| 27 | Practice | Do autistic people tend to follow the rules; e.g.; do not buy alcohol if you are under 18 or do they think they are able to make their own decisions about their alcohol consumption and do not need someone else to make a law about it? | 1. Do autistic people tend to follow the rules, e.g., do not buy alcohol if they are under 18 or do they think they are able to make their own decisions about their alcohol consumption and do not need someone else to make a law about it? 2. Do autistic individuals respond differently to current legislation/regulations around SABA? 3. Can some autistic features such as 'following rules' considered as strengths when aiming to reduce alcohol intake? 4. How to respond to the possible use of other than alcohol substances such as non-medical use of prescribed substances and illegal substances? |
| 28 | Research | What are the specific triggers; risk factors and facilitators of SABA in autistic people and what steps could be taken to prevent them? | 1. Is the age of diagnosis/recognition or identification of autism a predictor factor of SABA in later life? If autism isn't diagnosed early, if it's a late diagnosis, might that more likely predict SABAA in later life? 2. Does any of the following have an effect on addictions in autistic people: Eating well, use of probiotics, gluten and caffeine use, etc? 3. Do issues around gender identity and gender diversity have an effect on autistic people's SABA?' 4. What is the association between addiction, trauma and autistic ableism? 5. Does the intersection of being an autistic person and a part of a marginalized group, affect these individuals in a negative way so that it results in the process of starting using substances, having behavioural addictions or using alcohol in a problematic way? 6. What, if any, are the links between racial and intergenerational trauma and autistic people’s experiences and use of alcohol and substances? 7. What role do victimisation and stressful life events have in autistic peoples’ developing addictions? 8. Families’ and society’s understanding of autism and addictions, and the stigma around these conditions might have an impact on an individual’s mental health. How do these two conditions affect autistic people’s developing addictions? 9. Do autistic people use alcohol because they do not know enough about the potential negative effects of using it (in childhood) 10. What are the motivators of alcohol use in autistic individuals? 11. What made them start misusing alcohol? 12. Are neurotransmitters related to autism increase the likelihood of doing certain behaviours? 13. Are autistic people more prone to addiction? 14. What is the association between childhood neglect and problematic alcohol use in autistic individuals? Is this different from their neurotypical peers? 15. How does problematic alcohol use relate to camouflaging/ compensatory behavior? 16. Is stigma about autism a factor in why autistic people start to drink? 17. What are the differences between autistic individuals who have and do not have addictions? 18. what part does identity play in later alcohol use? 19. What is the role of peer pressure in problematic alcohol use of autistic individuals? 20. What is the role of living conditions such as living in a residency home that does not allow taking alcohol in the property, therefore encourages binge drinking off the property in problematic alcohol use of autistic people? 21. What role do self-worth or boredom play in problematic alcohol consumption in autistic individuals? 22. Is external locus of control common in autistic individuals and if so what kind of an impact it has on autistic individuals’ problem drinking? 23. Are they using alcohol as they are close to burnout, struggling to keep a job, marriage etc. 24. Do autistic individuals find it hard to express themselves and is this a factor that increases their alcohol consumption? 25. Can any of the triggering factors be preventable in autistic people with problematic alcohol use and if so, how? 26. Do any of the following have any impact on autism-substance use links? 27. cope with stress cope with social situations to fit in peer pressure expected to use such substances in that field/job e.g. banking they're not aware of the harms noise sensitivity + sleep 28. What is the role of perceived social awkwardness in substance use of autistic individuals? 29. Is there any association between substance use and any potential underlying trauma in autistic people? 30. Why do autistic individuals start drinking? 31. What research exists e.g. around social settings, potential social vulnerabilities being exploited in youth etc? 32. what is the role of peer influence and context on substance use? 33. Is emotional distress a cause of substance abuse in autistic people? 34. Do autistic people with substance use problems have an internal or external locus of control? 35. Is locus of control a factor in the substance use habits of autistic individuals? 36. are causal factors similar to those seen in non-autistic groups 37. Are the factors driving behavioral addictions similar or distinct from the reasons that individuals use alcohol or substances? |
| 29 | Research | What impact do other conditions or traits that autistic people may have (e.g. ADHD; anxiety; depression; OCD; impulse control etc) on the development and maintenance of SABA? | 1. How high is the risk of suicide and do they often feel themselves as a burden for their families? 2. Is alexithymia a factor that affects addictions in autistic people?' 3. What is the association between addiction, trauma and autistic ableism? 4. What, if any, are the links between racial and intergenerational trauma and autistic people’s experiences and use of alcohol and substances? 5. What role do life traumas (not limited to childhood) have in autistic people developing addictions? 6. What is the role of obsessive-compulsive traits in addictions in autistic people? 7. If there is a comorbid intellectual disability does that make any difference? 8. What percentage of autistic adults with problematic alcohol use have co-occurring mental health conditions? When were these diagnosed? How did their diagnosis affect their alcohol use? 9. Can diagnosis and treatment if co-occurring conditions in autistic people (such as anxiety/trauma/depression) help them to avoid problematic alcohol use in the first place? 10. Is problematic alcohol use related to impulse control in autistic people? 11. Can reducing anxiety that autistic individuals experience support autistic individuals in their alcohol recovery process? 12. Are autistic people and people with ADHD at high risk of having substance use disorder? 13. What is the role of learning disability in autism-problematic alcohol use association? 14. what % of addicted people are autistic, have ADD, and other neurodiverse conditions and have missed out on the help they need? 15. Does having other co-morbid neurodivergencies impact the likelihood of an autistic person having problematic alcohol use? 16. What is the role of co-occurring conditions such as dyslexia or learning disabilities in addiction? 17. Is there any association between substance use and any potential underlying trauma in autistic people? 18. what are ADHD, ASD and substance use associations? 19. what are the role of trauma and other underlying mental health concerns on substance use? 20. What percentage of autistic adults using substances have co-occurring mental health conditions? 21. What is the depression and/or anxiety, and substance use association in autistic people? 22. What are the comorbidities that autistic people have who use substances? 23. Are behavioural addictions transdiagnostic reflections of emotion regulation difficulties coping with stress and overwhelm? 24. Does alexithymia can explain why people with autism develop behavioural addictions 25. the underlying cause of "anxiety" in people with autism: is dysphoria related to substance misuse, underlying anxious personality, GAD... 26. Is gambling more or less prevalent among people with autism AND another substance misuse problem (co-morbidities)? |
| 30 | Research | What is the longitudinal course of SABA in autistic people and do these trajectories differ from those in non-autistic individuals? | 1. Does problematic alcohol use present in a similar or different way between people with autism and those who don't have autism? 2. Is there a greater conversion rate from substance use to substance use disorder in autistic people compared to the general population? 3. is there a difference between those who use substances other than alcohol? are there sensory factors involved? if they use one type of substance is it progressive or do they tend to stick to one? 4. Is there a greater conversion rate from substance use to substance use disorder in autistic people compared to the general population? 5. when does this start - why - does this follow a similar course to non-autistic people? |
| 31 | Research | How do neural pathways and executive functioning differ in autistic people who have SABA when compared to autistic people who do not have SABA and non-autistic people? | 1. How do neural pathways (along with the brain chemistry) work in autistic people who have addictions, especially behavioural addictions? 2. Are neurotransmitters related to autism increase the likelihood of doing certain behaviours? 3. Are the current treatments tailored to suit autistic individuals’ neurological needs? 4. How are neuropsychology and brain chemistry influences here? 5. Is an ASD brain wired to default to rapid dopamine release to salve the pain of communication difficulties? 6. Why do ASD individuals fail to recognise the risks of addiction despite being adept scientific/factual based brains? 7. Do ASD individuals’ neural pathways alter faster than neurotypical brains on substance exposure so their pathway to addiction is more rapid or are they less able to recognise their own reliance? 8. What is the role of executive functioning in the addiction-autism association? 9. Considering the research showing differences in the effects of psychedelics in this group, are there differences in the way that substances affect autistic people? Would be helpful to know about stimulants etc as this may then inform the discussion about why people get into problem substance use. 10. Is there a neurological difference between autistic individuals who use substances and autistic individuals who do not use substances 11. Is there any evidence on social reasons of substance use in autistic individuals? If there are, what are the ultimate results after neurological factors are controlled? 12. What are the neuropsychological differences between autistic people who have behavioural addictions and their neurotypical peers? |
| 32 | Research | What is the overlap between autism and SABA in terms of symptoms/features and comorbidity? | 1. Is one type of autism more likely to lead to addiction than another type? high functioning vs low functioning 2. Behavioural addictions is a relatively new research area compared to problematic alcohol use and substance use disorder. Does this explain the relatively small amount of research focused on behavioural addictions in autistic people? 3. What percentage of patients referred to behavioural addiction services have an underlying non-neurotypical brain? 4. What percentage of autistic people experience problematic alcohol use/ substance use or behavioural addictions compared to the general population? 5. Which features/symptoms overlap between these conditions; (between problematic alcohol use & autism, Substance use & autism and behavioural addictions & autism) 6. Is there any causal association between autism and substance use? 7. Is autism in children a reliable predictor of substance-related problems later in life? 8. How does their condition affect their psychology and the psychology of addiction? 9. What is the ratio of problematic alcohol use in autistic individuals? 10. Are autistic people more likely to suffer an addiction which contributes to their problematic alcohol use? 11. Do autistic individuals have problematic alcohol use? 12. Are autistic people more likely to suffer an addiction which contributes to their substance use? 13. Why is gambling disorder more prevalent among people with autism? What are the autism-specific factors? 14. What is the prevalence of behavioural addictions among autistic people? |
| 33 | Research | Is SABA a form of self-medication (e.g. to reduce diagnosed or undiagnosed anxiety) in autistic people? | 1. How does diagnosed or undiagnosed anxiety affect autistic individuals’ engagement with SABA as a form of self-medication? 2. Autistic people are more likely to use recreational substances to manage mental health-related problems including masking, compensation, and camouflaging. How that might impact on dependence? 3. Do autistic people drink alcohol as a coping mechanism? 4. Do autistic people drink to cope with feelings of isolation, loneliness, depression or anxiety? 5. Do they drink for self-medicating for anxiety states and problems with an underlying repetitive behavioural problem? 6. Is it a self-treatment strategy? 7. Are people with ASD more likely to self-medicate the feeling of being ‘outsiders’ or ‘other’ socially due to deficits in their social interactions - being bullied or marginalised creating feelings of inadequacy and low self-esteem? 8. Do people with autism find they are self-medicating a lack of social confidence initially - the anxiety caused by constant masking and camouflaging makes them use substances to ease the discomfort? 9. Do autistic people use alcohol to reduce sensory overload/ over-sensitivity? Is being in loud, crowded spaces with screens, flashing lights, music and people a risk factor for problematic alcohol use in autistic people? 10. What are the main ways of coping with stress for autistic people? 11. Are autistic people in pain because of their physical and mental health-related issues and do they use alcohol as a way of alleviating symptoms? 12. Is substances an emotional/social coping mechanism for autistic individuals? 13. How does substance use relate to chronic pain management? How does substance use relate to the eating behaviours of autistic adults? 14. Are behaviour addictions serve as a way of managing anxiety in autistic individuals? 15. Is the difficulty of trying any of these contribute to behavioural addictions in autistic people; trying to feel OK, connect socially and deal with sensory and emotional dysregulation? |
| 34 | Research | What associations are there between autistic features (e.g. emotion regulation; socializing difficulties; lower interoception; repetitive behaviours; rigid thinking; sensory issues; or special interests) and developing SABA? What is the nature of this interaction and what could be done to reduce negative effects? | 1. Autistic people are more likely to use recreational substances to manage mental health-related problems including masking, compensation, and camouflaging. How might that impact on dependence? 2. Does curiosity towards substances lead perhaps to develop an addiction later in life? Is it related to sensory-seeking behaviour, following other people saying it is a good or interesting sensory experience? 3. Is not recognising the feeling of being drunk due to it being an abstract concept makes autistic individuals unable to regulate their drinking? 4. Do autistic people rely on alcohol as a "safety blanket" when in social situations? 5. Is it because some autistic people have an 'all or nothing' approach, so have to finish a bottle? 6. Can restrictive routines impact on drinking - some people have created a rule about when or how much to drink (e.g. has to drink 2 bottles of wine as has to complete things in even numbers). 7. Is there any causal association between autism and substance use? 8. Are 'rigidity in thinking' or 'special interest' in substances, some of the features of autism phenotype, potential risk factors for autistic individuals who use substances in a problematic way? 9. How does alcohol serve as emotion regulation in autistic people? 10. Do autistic people use alcohol to reduce sensory overload/ over-sensitivity? Is being in loud, crowded spaces with screens, flashing lights, music and people a risk factor for problematic alcohol use in autistic people? 11. Do lower interoception and sensation seeking trigger alcohol use in autistic people by masking their understanding of how much is too much and what feels harmful? 12. Are any of these causing problematic alcohol use in autistic people?  - -a transdiagnostic overlap including self-harm, substances, addictive behaviours - -not being as adaptive to change - -emotion regulation difficulties - -have less emotional awareness  1. How does problematic alcohol use relate to camouflaging/ compensatory behaviour? 2. Do autistic individuals recognise incentives to start drinking? If they could recognise the incentives, would that reduce their problematic alcohol use? 3. How does inflexible thinking affect autistic individuals’ problematic alcohol use? Does it prevent them to see the 'other options'? 4. Are there any links between alcohol consumption, sleep and noise sensitivity in autistic adults? 5. What is the role of sensory overload in the development of both addiction and autism? 6. Does age at diagnosis (and masking) correlate with the number of autistic individuals with problematic alcohol use? 7. What is the role of perceived social awkwardness in substance use of autistic individuals? 8. What is the relationship between substances and sensory sensitivities? 9. How does substance use relate to camouflaging/ compensatory behaviour? 10. How do processing disorders work to form addictive habits? 11. Is this linked to other repetitive behaviours? 12. Do restricted preferences or difficulties with social interactions limit the availability of alternative sources of reinforcement, which in turn increase the probability of someone having an addiction? 13. Why is gambling disorder more prevalent among people with autism? What are the autism-specific factors? 14. What is the link between repetitive forms of addiction (such as gambling machines) and the repetitive behaviours seen in ASD? 15. how stimming behaviour interacts with behavioural addictions 16. Do they link to the individuals’ special interests, or ARE they the individuals’ special interests? 17. Do ASD traits of repetitive behaviours with inflexibility to change contribute to behavioural addictions? 18. Is there a link between repetitive behaviours and behavioural addictions in autistic people? 19. Can behavioural addictions be a source/form of "stimming"? 20. What is the role of autistic features such as preservation, executive function difficulties 'having a sticky brain' or black and white thinking in behavioural addictions in autistic people? Do they play a facilitating role? Do they accelerate addiction process compared to non-autistic people? 21. What is the role of special interests in behavioural addiction? 22. What is the role of repetitive behaviours in behavioural addiction (e.g., are individuals with higher RRBI scores more likely to develop behavioural addictions)? 23. Is the difficulty of trying any of these contribute to behavioural addictions in autistic people; trying to feel OK, connect socially and deal with sensory and emotional dysregulation? |
| 35 | Research | Is the presentation of SABA similar or different between autistic and non- autistic people? | 1. What percentage of patients referred to behavioural addiction services have an underlying non-neurotypical brain? 2. What percentage of autistic people experience problematic alcohol use/ substance use or behavioural addictions compared to the general population? 3. Are autistic people more or less likely to binge drink? 4. Are they likely to have atypical reactions to alcohol, as they sometimes do to medication, so they experience it differently? 5. Does problematic alcohol use present in a similar or different way between people with autism and those who don't have autism? 6. Is the pattern of denial stronger in people with ASD as they fail to recognise the harms to self or family? 7. What is the association between childhood neglect and problematic alcohol use in autistic individuals? Is this different from their neurotypical peers? 8. Do autistic individuals recognise incentives to start drinking? If they could recognise the incentives, would that reduce their problematic alcohol use? 9. Is there a difference between those who use substances other than alcohol? Are there sensory factors involved? If they use one type of substance is it progressive or do they tend to stick to one? 10. What is the impact of cannabis use in particular on people with autism compared to people without autism? 11. Is there a greater conversion rate from substance use to substance use disorder in autistic people compared to the general population? 12. Are autistic people more likely to suffer an addiction which contributes to their substance use? 13. When were these diagnosed? How did their diagnosis affect their substance use? How did the timing of their autism diagnosis affect their substance use? 14. Why is gambling disorder more prevalent among people with autism? What are the autism-specific factors? 15. Are the factors driving behavioural addictions similar or distinct from the reasons that individuals use alcohol or substances? |
| 36 | Research | What is the role of genetic predisposition and environment-gene interactions in autism and SABA (including any association between the two)? | 1. Given family history of alcohol use disorders might cause problematic alcohol use in later life in autistic individuals, what is the gene-environment interaction in autistic people who develop addictions? 2. Considering problematic alcohol use during the perinatal period and its outcomes in offspring (autistic traits in babies), is the mum’s drinking related to her autism, and is a baby autistic because it's genetic and coming from the mum? 3. Can genetics be an underlying factor for problematic alcohol use in autistic individuals? 4. Do a family history of alcohol use disorders have a role to play? 5. What are the role of genetic predisposition in autism and problematic alcohol use? 6. if there is any genetic basis or likelihood of affect family members. The IQ of affected individuals vs those with SUDs |
| 37 | Research | What impact do life stage transitions have on vulnerability to SABA in autistic people? | 1. How do life stage transitions affect autistic people and is there an association between particular difficult life stages and using substances, or having behavioural addictions or problematic alcohol use? 2. What role do life traumas (not limited to childhood) have in autistic people developing addictions? 3. Given family history of alcohol use disorders might cause problematic alcohol use in later life in autistic individuals, what is the gene-environment interaction in autistic people who develop addictions? 4. Are childhood traumas possible underlying vulnerabilities for autistic individuals who have problematic alcohol use 5. What are the perceptions of autistic people about the reasons other people use alcohol? and how much etc? 6. What is the association between childhood neglect and problematic alcohol use in autistic individuals? Is this different from their neurotypical peers? 7. Is the time of starting to drinking problematically tied to difficult periods/transitions? 8. what part does identity play in later alcohol use? 9. What is the role of living conditions such as living in a residency home that does not allow taking alcohol in the property, therefore encourages binge drinking off the property in problematic alcohol use of autistic people? 10. What impact do socializing and being a part of a peer group has on teenage substance abuse among autistic teenagers? 11. Is there any association between substance use and any potential underlying trauma in autistic people? 12. Is there any particular age that autistic individuals start to use substances? 13. Is there any link between using substances and childhood, parenting, schooling, bullying in autistic people? 14. What is the age of starting to use substances? Is there any peer group effect? 15. Are there particular life-events when someone with autism is more vulnerable to gambling disorder? |
| 38 | Research | Might autistic people engage in SABA as a form of positive reinforcement (e.g., excitement; arousal; social enhancement) | 1. Might autistic people engage in SABA as a form of positive reinforcement (e.g. excitement, arousal, social enhancement) 2. Does curiosity towards substances lead perhaps to develop an addiction later in life? Is it related to sensory seeking behaviour, following other people saying it is a good or interesting sensory experience? 3. Do autistic people drink alcohol to engage with others? 4. Do autistic people drink alcohol to appear 'non-autistic'? 5. Do autistic people drink to help them relax following anxiety provoking situations? 6. Do autistic people rely on alcohol as a "safety blanket" when in social situations? 7. What impact do socializing and being a part of a peer group has on teenage substance abuse among autistic teenagers? 8. Is using substances help autistic individuals to fit in social situations? 9. Do they link to the individuals special interests, ARE they the individuals special interest? |

1. <https://vimeo.com/630195200?ref=tw-share> [↑](#footnote-ref-1)
2. <https://twitter.com/pspsabaa> [↑](#footnote-ref-2)
3. Although we are aware of the fact that there is no universally agreed definition for autistic people, we use identity-first language on the SABAA website, publications and presentations to acknowledge that It is important to us as the study team to be as inclusive as possible of everyone who wishes to contribute and to be transparent in the processes, for this reason, we did not exclude or change the questions that might have come from people who may not be up to date with the latest ideas about the terminology and therefore use other terminologies to refer to autism. This lead to various terminologies used to refer to autism and/or autistic people in the raw survey data. However, as part of the 'translation' to the agreed final statements (Topic questions), we ensured that we used identity-first language to acknowledge the general preference of the autistic community. Please see Kenny and colleagues (2016) article for more information on autistic people’s preferences on the language (<https://journals.sagepub.com/doi/10.1177/1362361315588200?url_ver=Z39.88-2003&rfr_id=ori:rid:crossref.org&rfr_dat=cr_pub%20%200pubmed>). [↑](#footnote-ref-3)
